# An environmental scan of impacts and interventions for women with methamphetamine use in pregnancy and their children

**DOI:** 10.1002/ijgo.13851

**Published:** 2021-08-23

**Authors:** Melissa Ackerman, Claudia Madampage, Lynette J. Epp, Kali Gartner, Alexandra King

**Affiliations:** ^1^ Indigenous Wellness Research Group College of Medicine University of Saskatchewan Saskatoon Saskatchewan Canada; ^2^ College of Nursing University of Saskatchewan Saskatoon Saskatchewan Canada; ^3^ Department of Family Medicine University of Saskatchewan Saskatoon Saskatchewan Canada

**Keywords:** Infant/child outcomes, interventions, maternal outcomes, prenatal methamphetamine exposure, substance use in pregnancy

## Abstract

**Background:**

Indigenous women are overrepresented among people who use (PWU) methamphetamine (MA) due to colonialism and intergenerational trauma. Prenatal methamphetamine exposure (PME) is increasing as the number of PWUMA of childbearing age grows. Yet impacts of MA in pregnancy and effective interventions are not yet well understood.

**Objective:**

We conducted an environmental scan of published and grey literature (2010–2020) to determine effects of MA use in pregnancy for mothers and their offspring, effective interventions and implications for Indigenous women.

**Search strategy:**

A strategic search of Ovid Medline, Embase, ProQuest—Public Health and CINAHL databases identified academic literature, while Google and ProQuest—Public Health identified grey literature.

**Selection criteria:**

Article selection was based on titles, abstracts and keywords. The time frame captured recent MA composition and excluded literature impacted by coronavirus disease 2019.

**Data collection and analysis:**

Data extracted from 80 articles identified 463 results related to 210 outcomes, and seven interventions. Analysis focused on six categories: maternal, neonatal/infant, cognitive, behavioral, neurological, and interventions.

**Main results:**

Maternal outcomes were more congruent than child outcomes. The most prevalent outcomes were general neonatal/infant outcomes.

**Conclusion:**

A lack of Indigenous‐specific research on PME and interventions highlights a need for future research that incorporates relevant historical and sociocultural contexts.

## Synopsis

An environmental scan of published and grey literature (2010–2020) identified impacts of prenatal methamphetamine exposure for mothers and offspring, and identified promising interventions.

## 1 INTRODUCTION

Globally, Indigenous people, particularly women, are overrepresented among people who use (PWU) methamphetamine (MA).^1^, ^2^ This mirrors patterns of use for other licit and illicit substances, and is partially accounted for through consideration of colonization and associated trauma.^3^, ^4^, ^5^ Substance use is often identified as a coping strategy for personal and intergenerational trauma and mental health issues.^3^ Historic and sociocultural factors lead to additional and particular impacts of substance use on Indigenous peoples which amount to a “disproportionate burden of harm”.^1^ Health inequities experienced by Indigenous women are best addressed through approaches based on traditional knowledge, strengths‐based approaches, culture, and self‐determination.^6^

Substance use issues are gendered, with women experiencing different impacts, effects and outcomes around substance use compared with men, often due to socio‐contextual factors.^4^ In areas of Canada, rates of MA use are higher among women than men, and women start using MA at a younger age.^7^ Estimates suggest up to one‐third of people who use drugs are women of childbearing age.^3^, ^4^ Among incarcerated women in Canada, rates of substance use while pregnant are twice as high for Indigenous women than non‐Indigenous women.^5^ In addition, among people who use drugs, Indigenous people experience a higher incidence and prevalence of HIV compared with non‐Indigenous people,^8^ and injection drug use is a primary factor in new cases of HIV in Canada, particularly among Indigenous people.^9^ Despite colonial roots underpinning these statistics, societal discourse presents Indigenous people as an “at‐risk population”,^10^ whereby ethnicity is asserted as the determinant of health, as opposed to colonialism.^11^ As a result, stigma is compounded for those experiencing intersecting identities of pregnancy, substance use, HIV risk and Indigenous identity. Yet there is a lack of research and information on effects, treatments and access specific to women experiencing such complex life circumstances.^4^, ^12^

**FIGURE 1:**
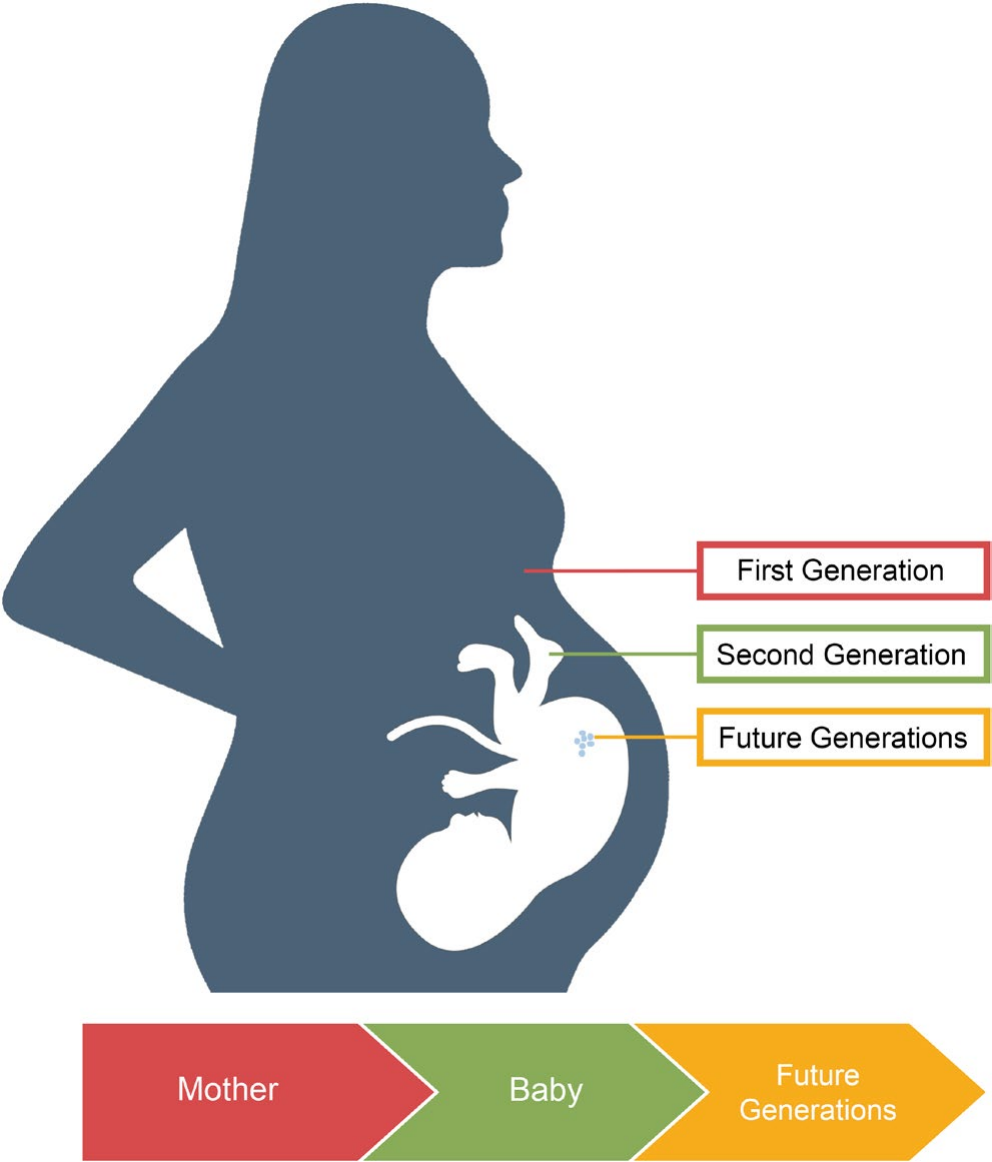
Impacts of methamphetamine use in pregnancy on multiple generations

MA is a central nervous system stimulant that has seen an extensive upsurge in number of users in the last few decades, particularly among women of childbearing age.^13^ Along with its growing prevalence, there is rising concern for prenatal methamphetamine exposure (PME) among women of childbearing age because of limited knowledge of effects of MA in pregnancy on women, their offspring and future generations.^13^ As illustrated in Figure 1, a Developmental Origins of Health and Disease lens demonstrates that effects of environmental stressors such as PME may transmit across several generations.^14^ This approach resonates with many Indigenous cultures, aligning with such concepts as Seven Generations, Blood Memory, and connectivity across families and generations.^15^

This environmental scan was designed to identify academic and grey literature on PME to summarize current knowledge on effects and interventions. Our aims for this scan emerged from our partnership with Sanctum 1.5 in Saskatoon, Saskatchewan, which is a 10‐bed residence that provides culturally safe, client‐centered wrap‐around care in a harm reduction context for perinatal women who are HIV positive or at risk of becoming positive due to factors including injection drug use.^16^ Over 80% of Sanctum 1.5 clients self‐identify as Indigenous, and many report using substances during their pregnancy, including MA. We identified academic and grey literature from 2010 to 2020 to answer the following:

1. What are the effects of MA use in pregnancy on mother and baby, particularly for women experiencing complex life circumstances?
2. What interventions can be useful to minimize or mitigate these effects?
3. What are the implications of the reviewed literature for Indigenous women, particularly those experiencing complex life circumstances such as clients at Sanctum 1.5?

## 2 MATERIALS AND METHODS

We conducted an environmental scan of academic and grey literature on effects of PME exposure on women and neonates/infants, and promising interventions. The final criteria for selected articles were:

- English language
- Research with human participants
- Published between January 1, 2010 and January 1, 2020.

Although we initially included articles from 2005 to 2020 in our search, we further restricted the date range to 2010–2020 to capture recent patterns in legal and health interventions for women who use substances and to reflect current drug composition to avoid confounding. For example, despite a decline in demand for MA from 2005 to 2008, the US observed reversing trends from 2009 to 2010, which corresponded to alterations in supply precursors transitioning from ephedrine and pseudoephedrine to recipes containing phenyl‐2‐propanone because of efforts to produce purer and more potent MA.^17^ Publications after January 1, 2020 were excluded because of the potential impacts of coronavirus disease 2019 on MA use and supply patterns.

Through consultation with co‐authors and a health sciences librarian, five databases were selected for our search: Ovid Medline, Embase, and CINAHL databases for academic literature, ProQuest—Public health for academic and grey literature, and Google for additional grey literature. As data were obtained from published and public sources, no research ethics review was required.

**FIGURE 2:**
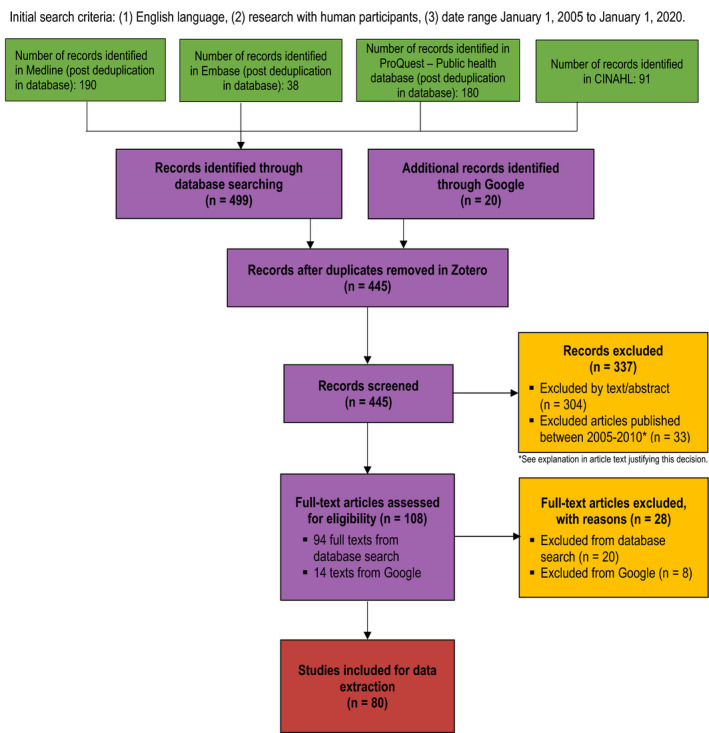
PRISMA chart depicting search protocol

The PRISMA chart in Figure 2 depicts our search protocol.^18^ Search terms originated from three main concepts: “intervention”, “methamphetamine”, and “pregnancy.” Due to preexisting awareness of the dearth of Indigenous‐specific health data;^19^ terms related to Indigeneity were not included in our search terms to capture a broader range of literature. Preliminary searches were conducted in Medline. “Intervention” was removed from search terms in subsequent academic databases, as a review of abstracts and titles from preliminary searches revealed that interventions were not well represented in this literature. Initial searches included “Amphetamine” and other relevant keywords and MeSH terms for prescription derivatives, as exploring the use of stimulant‐type prescriptions in pregnancy could provide further knowledge towards our objectives. However due to time constraints, “Amphetamine” and its related terms were later excluded from the search. Further review of titles and abstracts revealed that many results included MA use outside pregnancy, with most relevant results including terms such as “prenatal” or “prenatal exposure.” As such, terms that may not imply pregnancy such as “child,” “mothers,” “child, preschool” and “infant” were combined with “OR”, and then linked with pregnancy terms “pregnan*,” “prenat*,” and “pre‐nat*” using “AND”. The search was further narrowed by removing “postpartum” and “postnatal,” and “infant” and “child”, which do not explicitly reference pregnancy. Line‐by‐line strategies for academic and Google searches are available in Appendix S1.

Preliminary Google searches used the initial three concepts and an interchanging selection of synonyms. These results were reviewed for their relevance to the objectives. Although we included a complete list of synonyms and related terms in the search strategy for academic databases to ensure comprehensiveness, this strategy was less useful in Google. The final search term, Methamphetamine Pregnancy Approach, provided the most relevant results for our objectives. The first two pages of results were considered for inclusion, resulting in 10 sources—six of which were new, and four of which had been identified in the academic search.

Duplicate results were removed, and results from Medline, Embase, CINAHL and ProQuest were exported to zotero. Citations from Medline and Embase were combined into a spreadsheet containing these fields: database, title, author, MeSH subject headings, abstract, date of publication, URL, link to external resolver and keyword headings. Citations were also exported into rayyan, an online systematic review process tool. Within rayyan, articles were screened based on an initial review of their title, abstract, and use of keywords. At this point, 33 articles that met criteria in the initial screening and were published between 2005 and 2010 were excluded based on the impact of these articles and time constraints for this scan. As such, our date criteria were adjusted to articles published from 2010 to 2020. A total of 74 selected articles were cross‐referenced with the Medline and Embase citations and extracted into a final spreadsheet, which also included six results from Google. All 80 articles and annotations are listed in Appendix S2.

Annotations were written by the first author with the following components:

- Brief description of the article's purpose
- Relevance to the subject of interest if not explicitly expressed in the article's purpose
- Prominent findings/references
- Contrasting views where available.

Articles were also reviewed for results or conclusions specific to Indigenous people.

The annotations were catalogued into one of 10 categories according to main results:

1. Maternal/neonatal outcomes
2. Anthropometric measures
3. Visual outcomes
4. Behavioral outcomes
5. Cognitive outcomes
6. Case reports
7. Diffusion tensor imaging (DTI) studies
8. Neuroimaging studies
9. Interventions
10. Reviews

Annotations in each category were reviewed and where results also fit another category, they were included in both lists. We tallied the number of articles reporting a given outcome separately for articles that found an association with PME and articles that found no association.

The 10 categories were subsequently restructured into six: anthropometric measures were combined with general neonatal/infant outcomes; visual outcomes were included with cognitive outcomes; DTI study results were included with neuroimaging studies; and case reports and review articles were distributed into remaining categories according to the effects discussed. Finally, maternal and general infant outcomes were spilt into separate categories.

## 3 RESULTS

**FIGURE 3:**
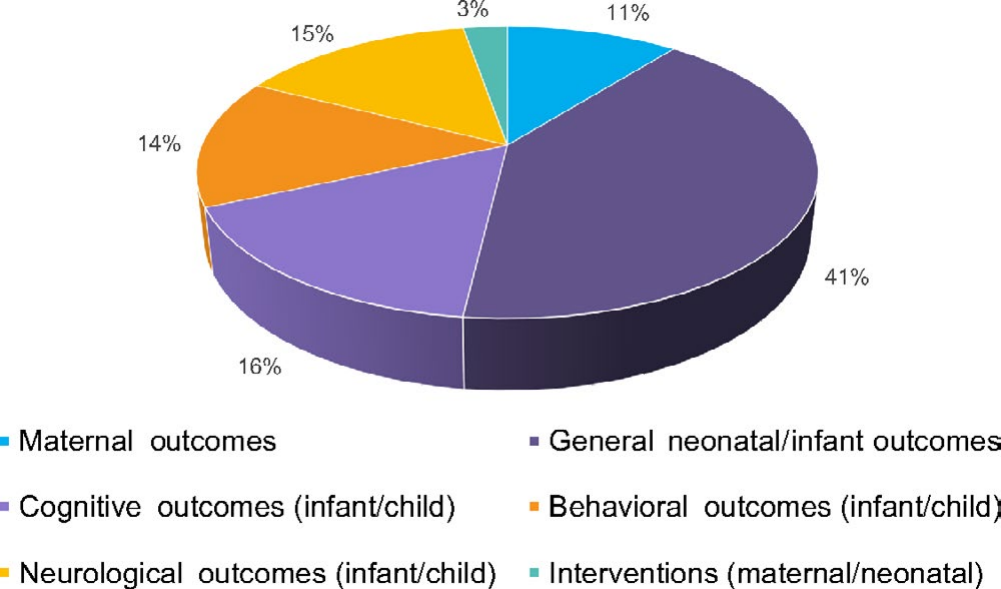
Distribution of results by category

The academic search identified 499 articles, 429 of which remained after de‐duplication. Using our criteria, 80 articles were identified and included in the results. Annotations were reviewed for specific outcomes, producing 463 “hits” related to 210 outcomes and seven interventions. Figure 3 outlines the distribution of these results among the final six categories discussed below. Outcome categories for each article are also identified in the “Output Codes” column in Appendix S2.

### 3.1 Maternal outcomes

Maternal outcomes associated with use of MA in pregnancy are listed in Table 1. Mothers were suggested to be at an increased risk of pre‐eclampsia, eclampsia and abruption, which were the most prevalent hypertensive diagnoses in the literature, with seven of eight articles (88%) reporting an association between prenatal MA use and hypertensive diseases. Other maternal outcomes included maternal weight gain and psychiatric disorders. The articles on maternal outcomes displayed more congruent evidence than literature related to infant/child outcomes.

**TABLE 1:** Maternal outcomes^a^

Maternal effects	Articles reporting an association with PME (*n*)	Articles reporting no association with PME (*n*)
Blood‐related conditions		
Abruption	(*n* = 5) Chatterjee (2018); Gorman et al. (2014); Louw (2018); Madide et al. (2012); Smid (2017)	
Anemia	(*n* = 1) Gargari et al. (2012)	
Hemorrhages	(*n* = 1) Gargari et al. (2012)	
Complications		
Complications		(*n* = 1) Smith et al. (2015)
Delivery		
Cesarean delivery	(*n* = 1) Smid (2017)	
Diabetes‐related conditions		
Gestational diabetes	(*n* = 1) Gorman et al. (2014)^b^	
Weight gain during pregnancy	(*n* = 3) Chang et al. (2016); Nguyen et al. (2010); Zabaneh et al. (2012)	
Hypertensive‐related conditions		
Eclampsia	(*n* = 2) Chatterjee (2018); Gorman et al. (2014)	
Hypertensive diseases	(*n* = 7) Dinger et al. (2017); Forray et al. (2015); Gorman et al. (2014); Louw (2018); Madide et al. (2012); Padilla et al. (2017); Smid (2017)	(*n* = 1) Kalaitzopoulos et al. (2018)
Pre‐eclampsia	(*n* = 6) Forray et al. (2015); Dinger et al. (2017); Gorman et al. (2014); Louw (2018); Madide et al. (2012); Smid, M. (2017)	(*n* = 1) Kalaitzopoulos et al. (2018)
Impaired function		
Below optimal functioning while under the influence of MA	(*n* = 1) Madide et al. (2012)	
Lifelong effects		
Risk of acquiring lifelong complications: blood transfusion, heart failure, cardiac arrest, other cardiac related concerns	(*n* = 2) Chatterjee (2018); Smid (2017)	
Structural brain changes (i.e., white‐matter reduction and deep‐brain strokes)	(*n* = 1) Madide et al. (2012)	
Mental illness/disorders		
Aggressive paranoid psychosis	(*n* = 1) Madide et al. (2012)	
Altered behavior	(*n* = 1) Madide et al. (2012)	
Cognitive impairments	(*n* = 1) Madide et al. (2012)	
Comorbidity of SUD and a positive diagnosis of a psychiatric disorder	(*n* = 2) Wouldes (2013); Wouldes (n.d)	
Emotional deficits	(*n* = 1) Madide et al. (2012)	
Psychiatric disorder/emotional illness	(*n* = 2) Shah (2012); Wouldes et al. (2013)	
Stress	(*n* = 1) Liles et al. (2012)	
SUD	(*n* = 1) Wouldes et al. (2013)	
Mortality		
Death during or after pregnancy	(*n* = 1) Chatterjee (2018)	
Pregnancy loss		
Terminated pregnancies (e.g., abortion)	(*n* = 2) Brecht et al. (2014); Dinger et al. (2017)	
Sexually transmissible and blood‐borne infections		
Gonorrhea	(*n* = 1) Shah et al. (2012)	
Risk for acquiring hepatitis B and/or C	(*n* = 1) Madide et al. (2012)	
Risk for acquiring HIV	(*n* = 1) Madide et al. (2012)	
Risk for acquiring sexually transmitted infections	(*n* = 1) Madide et al. (2012)	

Abbreviations: PME, prenatal methamphetamine exposure; SUD, substance use disorder.

^a^ Articles referenced in this table are cited in full in Appendix S2: Annotations.

^b^ Suggests PME is protective against acquiring gestational diabetes.

### 3.2 General neonatal/infant outcomes

These outcomes, listed in Table 2, represent the most prevalent outcomes in children with PME and include various anthropometric measures and neonatal/infant features. The most frequently reported anthropometric measures associated with PME included birth weight/small infant (discussed in 18 articles), head circumference (discussed in 10 articles), and body length (discussed in 11 articles). The association between PME and low birth weight was supported by a majority of articles (*n* = 13, 72%), while five articles (28%) negated the relationship. Similarly, most articles (*n* = 9, 90%) found that PME was linked to a decrease in head circumference, while one article (*n* = 1, 10%) refuted this association. Other frequently discussed concerns involved preterm birth/shorter gestational age, abnormal reflexes/poor quality of movement, and increased stress. One study reported nation‐dependent changes in anthropometric measures, which they attributed to differences in government policies and support.^20^

**TABLE 2:** General neonatal/infant outcomes^a^

Neonatal/infant effects	Articles reporting an association with PME (*n*)	Articles reporting no association with PME (*n*)
Anthropometric changes		
Anthropometric measures		(*n* = 1) Roos et al. (2015)
Birth weight <2500 g		(*n* = 1) Zabaneh et al. (2012)
Decreased body length	(*n* = 11) Abar et al. (2014); Gargari et al. (2012); Kalaitzopoulos et al. (2018); Kiblawi et al. (2013); Kiblawi et al. (2014); LaGasse et al. (2011); Ross et al. (2014); Shah et al. (2012); Smith et al. (2011); Wouldes et al. (2014); Wright et al. (2015)	
Lower birth weight/small infant	(*n* = 13) ACOG Committee (2011); Canadian Centre on Substance Use and Addiction (2018); Chatterjee (2018); Dinger et al. (2017); Forray et al. (2015); Gargari et al. (2012); Gorman et al. (2014); Hayward et al. (2010); Kalaitzopoulos et al. (2018); Louw (2018); Madide et al. (2012); Ross et al. (2014); Wouldes et al. (2014); Wright et al. (2015)	(*n* = 5) Abar et al. (2014); Nguyen et al. (2010); Ross et al. (2014); Smith et al. (2011); Zabaneh et al. (2012)
Macrosomia	(*n* = 1) Gorman et al. (2014)^d^	
Smaller head circumference	(*n *= 9) Dinger et al. (2017); Gargari et al. (2012); Kalaitzopoulos et al. (2018); Kiblawi et al. (2014); LaGasse et al. (2011); Madide et al. (2012); Ross et al. (2014); Shah et al. (2012); Smith et (2011); Wouldes et al. (2014)	(*n* = 1) Ross et al. (2014)
Apgar score		
Low Apgar score	(*n* = 6) ACOG Committee (2011); Good et al. (2010); Kalaitzopoulos et al. (2018); Kiblawi et al. (2014); Madide et al. (2012); Smith et al. (2012)	
Birth defects		
Birth defects		(*n* = 1) ACOG Committee (2011)
Cardiovascular complications/defects	(*n* = 2) Perumal et al. (2019); Pierce et al. (2019)	(*n* = 2) Dinger et al. (2017); Smith et al. (2016)
Congenital deformities	(*n* = 2) Chamberlain (2015); Smid et al. (2019)	(*n* = 3) Behnke et al. (2013); Dinger et al. (2017); Smid et al. (2019)
Congenital nystagmus	(*n* = 2) Goldberg et al. (2010); Heiss et al. (2010)	
Facial dysmorphism		(*n* = 1) Smith et al. (2016)
Pale retinae (ocular albinism)	(*n* = 2) Goldberg et al. (2010); Heiss et al. (2010)	
Respiratory problems		(*n* = 1) Smith et al. (2016)
Skeletal defects		(*n* = 1) Smith et al. (2016)
Blood‐related conditions		
Anemia	(*n* = 2) Goldberg et al. (2010); Heiss et al. (2010)	
Coagulopathy	(*n* = 1) Maranella et al. (2019)	
Thrombocytopenia	(*n* =1) Maranella et al. (2019)	
Brain‐related outcomes		
Acute neurological symptoms; i.e., encephalopathy, diffuse white‐matter damage, ischemic‐hemorrhagic lesions, hypotonia and spontaneous motor activity on the left side	(*n* = 1) Maranella et al. (2019)	
Agenesis of foramen of Monro and cortical maldevelopment (complex brain malformation)	(*n* = 1) Maya‐Enero et al. (2018)	
Brain abnormalities in fetus	(*n* = 1) Canadian Centre on Substance Use and Addiction (2018)	
Cerebral palsy	(*n* = 2) Goldberg et al. (2010); Heiss et al. (2010)	
Complex partial epilepsy	(*n* = 2) Goldberg et al. (2010); Heiss et al. (2010)	
Hydranencephaly	(*n* = 1) Cardwell (2014)	
Leukomalacia	(*n* = 1) Maranella et al. (2019)	
Right thalamic infarct in utero with left hemiparesis including the face	(*n *= 2) Goldberg et al. (2010); Heiss et al. (2010)	
Catecholamine resistance		
Catecholamine resistance	(*n* = 1) Perumal et al. (2019)	
Child Protective Services involvement		
CPS involvement	(*n* = 1) Shah et al. (2012)	
Dietary consumption		
Swallowing		(*n* = 1) Goldberg et al. (2010)
Transitioning to solid food	(*n* = 1) Goldberg et al. (2010)	
Gender		
Gender		(*n* = 2) Smith et al. (2011); Zabaneh et al. (2012)
Growth patterns		
Growth rate per month by age 3		(*n* = 1) Zabaneh et al. (2012)
Shorter height trajectory over the first 3 years from birth	(*n* = 1) Zabaneh et al. (2012)	
Weight‐for‐length growth trajectories		(n = 2) Ross et al. (2014); Zabaneh et al. (2012)
Heart‐related outcomes		
Heart abnormalities in fetus	(*n* = 1) Canadian Centre on Substance Use and Addiction (2018)	
Intrauterine growth restriction		
Intrauterine growth restriction/growth restriction	(*n* = 6) Louw (2018); Madide et al. (2012); The Royal Women's Hospital (2017); Ross et al. (2014); Smid (2017); Smith et al. (2016)	
Small for gestational age	(*n* = 6) ACOG Committee (2011); Cardwell (2014); Chatterjee (2018); Gorman et al. (2014); Madide et al. (2012); Nguyen et al. (2010)	(*n* = 3) Smith et al. (2011); Wright et al. (2015); Zabaneh et al. (2012)
Liver conditions		
Acute hepatotoxic symptoms; i.e. hepatic insufficiency	(*n* = 1) Maranella et al. (2019)	
Meconium aspiration		
Risk of intrauterine passing of meconium	(*n* = 1) Warton, Meintjes et al. (2018)	
Mortality		
Infant death (term born)	(*n* = 3) Dinger et (2017); Gorman et al. (2014); Louw (2018)	
Intrauterine fetal death	(*n* = 5) Brecht et al. (2014); Dinger et al. (2017); Forray et al. (2015); Gorman et al. (2014); Louw et al. (2018)	
Neonate death (term born)	(*n* = 6) ACOG Committee (2011); Dinger et al. (2017); Good et al. (2010); Louw (2018); Madide et al. (2012); Smid et al. (2019)	(*n* = 1) Gorman et al. (2014)
Stillborn	(*n* = 1) Sakai et al. (2015)	
Muscle tone		
Delayed development on active muscle tone	(*n* = 1) Chang et al. (2016)	
Delayed development on active muscle tone and total Amiel‐Tison neurological assessment at term (ATNAT) scores	(*n* = 1) Chang et al. (2016)	
Neonatal withdrawal		
Abnormal reflexes/poor quality of movement	(*n* = 8) ACOG Committee (2011); Behnke et al. (2013); Chamberlain (2015); Dinger et al. (2017); LaGasse et al. (2011); Roussotte et al. (2010); Smith et al. (2016); Wouldes (n.d)	
Arousal/excitability	(*n* = 8) ACOG Committee (2011); Behnke et al. (2013); Dinger et al. (2017); LaGasse et al. (2011); Roussotte et al. (2010); Smid (2017); Smith et al. (2012); Zuloaga et al. (2015)	(n = 1) Kiblawi et al. (2014)
Decreased ability to self‐regulate	(*n* = 1) Smith et al. (2012)	
Difficulty feeding	(*n* = 1) Louw 2018	
Excessive sucking	(*n* = 1) Dinger et al. (2017)	
Extreme irritability	(*n* = 1) Chamberlain (2015)	
Impaired arousal from sleep		(*n* = 1) Galland et al. (2013)
Increased handling	(*n* = 1) Smith et al. (2012)	
Increased lethargy and hypotonicity	(*n* = 1) LaGasse et al. (2011)	
Neonatal abstinence syndrome	(*n* = 2) The Royal Women's Hospital (2017); Wouldes, Lester et al. (2019)	(*n* = 3) Gargari et al. (2012); Smith et al. (2015); Smith et al. (2016)
Newborn jitteriness	(*n* = 1) Dinger et al. (2017)	
Poor suck	(*n* = 2) Dinger et al. (2017); Shah et al. (2012)	(*n* = 1) Goldberg et al. (2010)
Stress: total stress, more physiological stress, more CNS stress; increased physiological stress (neonate)	(*n* = 8) ACOG Committee (2011); Behnke et al. (2013); Dinger et al. (2017); Kiblawi et al. (2014); LaGasse et al. (2011); Madide et al. (2012); Smith et al. (2015); Smith et al. (2016)	(*n* = 1) Kiblawi et al. (2014)
Thrush after birth	(*n* = 1) Goldberg et al. (2010)	
"Weak" cry after birth	(*n* = 1) Goldberg et al. (2010)	
Newborn‐related health outcomes		
Newborn health outcomes		(*n* = 1) Smith et al. (2015)
NICU admission		
Admission to NICU/ICU	(*n* = 4) Gargari et al. (2012); Louw (2018); Shah et al. (2012); Smid (2017)	
Preterm delivery		
Preterm birth/shorter gestation	(*n* = 20) Canadian Centre on Substance Use and Addiction (2018); Chamberlain (2015); Chatterjee (2018); Dinger et al. (2017); Forray et al. (2015); Gargari et al. (2012); Good et al. (2010); Gorman et al. (2014); Gregory et al. (2017); Kalaitzopoulos et al. (2018); Kiblawi et al. (2013); Kiblawi et al. (2014); LaGasse et al. (2011); Louw (2018); Madide et al. (2012); Smid (2017); Smith et al. (2011); The Royal Women's Hospital (2017); Warton, Meintjes et al. (2018); Wright et al. (2015)	
Teratogenicity		
Teratogenicity		(*n* = 1) Dinger et al. (2017)

Abbreviations: CNS, central nervous system; CPS, Child Protective Services; ICU, intensive care unit; NICU, neonatal intensive care unit; PME, prenatal methamphetamine exposure; SUD, substance use disorder.

^a^ Articles referenced in this table are cited in full in Appendix S2: Annotations.

^b^ Suggests PME is protective against the occurrence of macrosomia.

### 3.3 Cognitive outcomes (infant/child)

Table 3 contains an index of cognitive changes (including visual outcomes) found in children with PME. Affective outcomes including anxiety, depression, emotional reactivity, aggression and/or affective problems represent one of the most frequently reported cognitive effects in children with PME. “Verbal/visual memory”, and “visual motor integration/low age equivalent score for visual development” were also frequently discussed. Several studies indicated an increase in cognitive problem subscale scores and an increased likelihood for above average cognitive problems in children with PME. Other less frequently identified changes included alterations in IQ, gross motor performance/low age equivalent score on gross motor development, reductions in fine motor development and performance, impacts on long‐term spatial memory, psychosomatic issues, and higher stress‐induced cortisol levels. Literature specific to fine motor coordination was contradictory, with one (50%) contending that PME was associated with this outcome, and the other (50%) negating the relationship. Our findings revealed a paucity of data regarding visual outcomes. Some visual features, such as global motion perception, habitual visual acuity, and stereopsis, were not found to be associated with PME. Some articles reported associations between visual motor integration and visual development and PME.

**TABLE 3:** Cognitive outcomes (infant/child), including visual outcomes^a^

Cognitive effects	Articles reporting an association with PME (*n*)	Articles reporting no association with PME (*n*)
Cognitive problems		
Cognitive control	(*n* = 1) Kwiatkowski et al. (2018)	
Higher cognitive problem subscale scores/increased likelihood for above average cognitive problems scores	(*n* = 5) Diaz et al. (2014); Dinger et al. (2017); Goldberg et al. (2010); Heiss et al. (2010); Louw (2018)	
Cognitive function/performance		
Cognitive scores on BSID‐II		(*n* = 1) Wouldes et al. (2014)
Heavy use associated with poorer overall neurodevelopmental function	(*n* = 1) Smith et al. (2016)	
Mental development index (BSID‐II)		(*n* = 2) Lester et al. (2010); Smith et al. (2011)
Neurocognitive performance	(*n* = 1) Madide et al. (2012)	
Poorer on cognitive functioning	(*n* = 1) Smid (2017)	
Psychomotor scores on BSID‐II/psychomotor performance	(*n* = 1) Wouldes et al. (2014)	(*n* = 1) Smith et al. (2011)
IQ		
Below normal intelligence age score	(*n* = 1) Heiss et al. (2010)	
IQ	(*n* = 4) Brinker et al. (2019); Dinger et al. (2017); Gutwinkski et al. (2017); Kwiatkowski et al. (2018)	(*n* = 1) Piper et al. (2011)
Processing speed (IQ)	(*n* = 1) Brinker et al. (2019)	
Memory		
Confrontation naming ability	(*n* = 1) Kwiatkowski et al. (2018)	
Spatial memory/lower scores on long‐term spatial memory	(*n* = 3) ACOG Committee (2011); Piper et al. (2011); Ross et al. (2014)	
Verbal/or visual memory	(*n* = 6) ACOG Committee (2011); Kwiatkowski et al. (2014); Kwiatkowski et al. (2018); Madide et al. (2012); Ross et al. (2014); Roussotte et al. (2010)	
Working memory	(*n* = 1) Kwiatkowski et al. (2018)	
Mental health		
Higher stress‐induced cortisol levels	(*n* = 2) Kirlic et al. (2013); Zuloaga et al. (2015)	(*n* = 1) Kirlic et al. (2013)
Increased anxiety/depression (child), and/or emotionally reactive, and/or aggressive, and/or affective problems	(*n* = 6) Abar et al. (2013); Dyk et al. (2014); LaGasse et al. (2012); Smid (2017); Smith et al. (2016); Zuloaga et al. (2015)	(*n* = 1) LaGasse et al. (2012)
Psychosomatic issues/somatic complaints	(*n* = 3) Dyk et al. (2014); Heiss et al. (2010); LaGasse et al. (2012)	
Transgenerational effects (i.e., resilience)	(*n* = 1) Dinger et al. (2017)	
Metabolic‐related cognitive changes		
Metabolic correlates to cognition	(*n* = 1) Kwiatkowski et al. (2014)	
Metacognition		
Metacognition	(*n* = 1) Piper et al. (2011)	
Motor performance		
Decreased fine motor development on PDMS‐II/ low age equivalent score on fine motor development/low fine motor performance	(*n* = 3) Heiss et al. (2010); Ross et al. (2014); Smith et al. (2015)	(*n* = 1) Wouldes et al. (2014)
Fine motor coordination	(*n* = 1) Smith et al. (2011)	(*n* = 1) Kwiatkowski et al. (2018)
General performance	(*n* = 1) Dyk et al. (2014)	
Gross motor performance/low age equivalent score on gross motor development	(*n* = 4) Dinger et al. (2017); Gutwinkski et al. (2017); Heiss et al. (2010); Wouldes et al. (2014)	
Mediating role of gender and exposure outcome (fine motor and cognitive performances.)	(*n* = 1) Wouldes et al. (2014)	
School‐aged learning		
Learning ability	(*n* = 1) Kwiatkowski et al. (2018)	
Behind classmates in reading	(*n* = 1) Piper et al. (2011)	
Behind children of similar age in school	(*n* = 1) Piper et al. (2011)	
Below average language skills/language development—limited vocabulary, but age‐appropriate speech production	(*n* = 1) Heiss et al. (2010)	(*n* = 1) Smith et al. (2016)
Lower school performances in math and language at 14 years	(*n* = 2) Dinger et al. (2017); Gutwinkski et al. (2017)	
Vision		
Eye and hand coordination	(*n* = 1) Dyk et al. (2014)	
Global motion perception		(*n* = 1) Chakraborty et al. (2015)
Habitual visual acuity		(*n* = 1) Chakraborty et al. (2015)
Stereopsis		(*n* = 1) Chakraborty et al. (2015)
Visual motor integration/low age equivalent score for visual development	(*n* = 7) ACOG Committee (2011); Colby et al. (2012); Heiss et al. (2010); Kwiatkowski et al. (2018); Ross et al. (2014); Roussotte et al. (2010); Smith (2017)	

Abbreviations: BSID‐II, Bayley Scales of Infant Development, Second Edition; PDMS‐II, Peabody Developmental Motor Scale, Second Edition; PME, prenatal methamphetamine exposure.

^a^ Articles referenced in this table are cited in full in Appendix S2: Annotations.

### 3.4 Behavioral outcomes (infant/child)

Table 4 includes a list of behavioral outcomes studied in children with PME. Attention‐deficit/hyperactivity disorder (ADHD) was the most commonly discussed behavioral effect associated with this population with nine of ten articles (90%) reporting a link between ADHD and prenatal exposure to MA. Similarly, of eight articles reporting results related to attention, all eight (100%) reported a relationship between decreased attention/inattention and PME. Other findings included altered executive function, externalization, aggressive behavior, and neurobehavior/neurobehavioral differences. Both internalizing and total behavioral problems had divided results each had one study supporting and one study negating the relationship between each respective outcome and PME.

**TABLE 4:** Behavioral outcomes (infant/child)^a^

Behavioral effects	Articles reporting an association with PME (*n*)	Articles reporting no association with PME (*n*)
ADHD‐related changes		
Attention‐deficit/hyperactivity disorder index; hyperactivity; ADHD; increased ADHD confidence score	(*n* = 9) Diaz et al. (2014); Dyk et al. (2014); Goldberg et al. (2010); Heiss et al. (2010); Kiblawi et al. (2013); Kirlic et al. (2013); LaGasse et al. (2012); Piper et al. (2011); Smith et al. (2016)	(*n* = 1) Diaz et al. (2014)
Behavioral control	(*n* = 3) Abar et al. (2013); Kwiatkowski et al. (2018); Piper et al. (2011)	
Decreased attention/inattention	(*n* = 8) ACOG Committee (2011); Derauf, Lester et al. (2012); Heiss et al. (2010); Kwiatkowski et al. (2018); Madide et al. (2012); Ross et al. (2014); Smid (2017); Smith et al. (2017)	
Externalization	(*n* = 5) Diaz et al. (2014); Eze et al. (2016); Ross et al. (2014); Smith et al. (2016); Twomey et al. (2013)	
Impulsivity	(*n* = 1) Heiss et al. (2010)	
Rule‐breaking behavior	(*n* = 2) Eze et al. (2016); Smith et al. (2016)	
Vigilance		(*n* = 1) Piper et al. (2011)
Aggression		
Aggressive behavior	(*n* = 5) Abar et al. (2013); Dinger et al. (2017); Dyk et al. (2014); LaGasse et al. (2012); Smith et al. (2016)	
Behavioral problems		
Poorer neurobehavior/ neurobehavioral differences	(*n* = 5) Behnke et al. (2013); Hayward et al. (2010); Madide et al. (2012); Roussotte et al. (2010); Wouldes, Lester et al. (2019)	(*n* = 1) Madide et al. (2012)
Risk of behavioral effects	(*n* = 1) Louw et al. (2018)	
Total behavioral problems	(*n* = 1) Twomey et al. (2013)	(*n* = 1) LaGasse et al. (2012)
Coping‐related behavior		
Compulsive eating tendencies	(*n* = 1) Heiss et al. (2010)	
High adversity index scores	(*n* = 1) Eze et al. (2016)	
Internalizing	(*n* = 1) Twomey et al. (2013)	(*n* = 1) LaGasse et al. (2012)
Personal and/or social	(*n* = 2) Dyk et al. (2014); Heiss et al. (2010)	
Withdrawn	(*n* = 1) LaGasse et al. (2012)	
Development		
Altered neonatal behavior	(*n* = 1) Chamberlain et al. (2015)	
Developmental and behavioral deficits	(*n* = 1) Forray et al. (2015)	
Pervasive developmental problems	(*n* = 1) Dyk et al. (2014)	
Executive function		
Executive function	(*n* = 5) Abar et al. (2013); Himes et al. (2014); Kwiatkowski et al. (2018); Piper et al. (2011); Twomey et al. (2013)	
Global executive composite	(*n* = 1) Piper et al. (2011)	
Poorer inhibitory control associated with heavy exposure	(*n* = 2) Derauf, LaGasse et al. (2012); Smith et al. (2016)	
Metabolic‐related changes		
Metabolic correlates to behavior	(*n* = 1) Kwiatkowski et al. (2014)	
Parental influence		
Maternal perception of child behavior problems		(*n* = 1) Liles et al. (2012)
Parental stress and psychological symptoms influence behavioral problems	(*n* = 1) Dinger et al. (2017)	

Abbreviations: ADHD, attention deficit hyperactivity disorder; PME, prenatal methamphetamine exposure.

^a^ Articles referenced in this table are cited in full in Appendix S2: Annotations.

### 3.5 Neurological outcomes from neuroimaging studies (infant/child)

Neurological outcomes investigated by neuroimaging modalities are cataloged in Table 5, and include brain circuitry and structure, brain volume changes, and striatal structure changes. The relationship between PME and altered brain structures was inconclusive, with one review article reporting mixed results for this relationship.

**TABLE 5:** Neurological outcomes from neuroimaging studies (infant/child)^a^

Neurological effects	Articles reporting an association with PME (*n*)	Articles reporting no association with PME (*n*)
Brain activation and function		
Ability to adjust to changing task demands (K‐CPT reaction time by inter‐stimulus interval)	(*n* = 1) Derauf, Lester et al. (2012)	
Less activation in left hemisphere: including the middle frontal gyrus, the precentral gyrus, the frontal orbital cortex, the superior and middle temporal gyri, the temporal pole, the palnum temporale and insula	(*n* = 1) Roussotte et al. (2011)	
More diffuse brain activation during verbal memory tasks	(*n* = 2) ACOG Committee (2011); Roussotte et al. (2010)	
Negative correlation between performance and activation in: inferior temporal gyrus, temporal pole, middle temporal gyrus, the anterior cingulate and paracingulate gyri, the frontal orbital cortex, and frontal pole bilaterally and the left superior frontal gyrus	(*n* = 1) Roussotte et al. (2011)	
Brain circuitry and structure		
Altered brain structures	(*n* = 1) Kwiatkowski et al. (2014)	(*n* = 1) Kwiatkowski et al. (2014)
Altered neural circuitry	(*n* = 2) Madide et al. (2012); Morie et al. (2019)	
HPA alterations linked to changes	(*n* = 1) Zuloaga et al. (2015)	
Metabolic changes	(*n* = 1) Roussotte et al. (2010)	
Microstructural brain changes	(*n* = 1) Kwiatkowski et al. (2014)	
Brain development		
Impaired brain development	(*n* = 2) Dinger et al. (2017); Tsai et al. (2019)	
Neurologic development	(*n* = 1) Roos et al. (2016)	
Brain injuries		
Brain cavitation and infarction, commonly involving the basal ganglia, deep in the frontal lobes and posterior fossa	(*n* = 1) Madide et al. (2012)	
Brain hemorrhage	(*n* = 1) Madide et al. (2012)	
Echolucent lesions	(*n* = 1) Madide et al. (2012)	
Periventricular leukomalacia and subsequent spastic quadriplegia		(*n* = 1) Madide et al. (2012)
Brain volume changes		
Brain volume	(*n* = 2) Burger et al. (2015); Kwiatkowski et al. (2014)	
Greater increase in volume in the anterior and posterior cingulate regions and the left and right perisylvian cortices (compared with alcohol‐exposed group)	(*n* = 1) Sowell et al. (2010)	
Reduced mid‐posterior corpus callosum volume (in exposed females compared with control females)	(*n* = 1) Roos et al. (2014)	
Reduction in the prefrontal lobe	(*n* = 1) Gutwinski et al. (2017)	
Volume reduction in left occipitoparietal cortices	(*n* = 1) Madide et al. (2012)	
Volume reduction in right prefrontal cortices	(*n* = 1) Madide et al. (2012)	
Caudate changes		
Fewer negative correlations between caudate seeds and occipital regions	(*n* = 1) Roussotte et al. (2012)	
Less activation in brain areas: frontal and basal ganglia regions in the left hemisphere during working memory, most prominent in left caudate, left putamen, and left inferior frontal gyrus, around Broca's area	(*n* = 1) Roussotte et al. (2011)	
Reduced connection between the dorsal caudate and frontal executive network	(*n* = 1) Roussotte et al. (2012)	
Caudate volume changes		
Negative association between neurocognitive scores and caudate volume	(*n* = 2) Madide et al. (2012); Sowell et al. (2010)	
Reduced left caudate volume		(*n* = 1) Warton, Meintjes et al. (2018)
Reduced right caudate volume	(*n* = 1) Warton, Meintjes et al. (2018)	
Volume reductions in caudate	(*n* = 4) Ross et al. (2014); Roussotte et al. (2010); Smith (2017); Sowell et al. (2010)	
Cortical thickness changes		
Greater cortical thickness in superior central sulcus and cuneus (in exposed females when compared with control females)	(*n* = 1) Roos et al. (2014)	
Increases in cortical thickness in perisylvian and orbital‐frontal cortices	(*n* = 1) Ross et al. (2014)	
Reduced left hemisphere cortical thickness of the inferior parietal, parsopercularis and precuneaus	(*n* = 1) Roos et al. (2014)	
Volume reductions in cortical and subcortical brain structures	(*n* = 1) Dinger et al. (2017)	
Globus pallidus changes		
Reduction in subcortical region of globus pallidus; associated with delayed verbal memory and poor sustained attention	(*n* = 1) Madide et al. (2012)	
Smaller globus pallidus and putamen; associated with attentional task	(*n* = 2) Kwiatkowski et al. (2014); Madide et al. (2012)	
Hippocampus changes		
Reduction in subcortical region of the hippocampus; associated with delayed verbal memory and poor sustained attention	(*n* = 1) Madide et al. (2012); Smith (2017)	
Reduced volume in hippocampus; associated with poorer performance on attention and memory tasks; behavior was also linked with reduced hippocampus	(*n* = 2) ACOG Committee (2011); Madide et al. (2012)	
Limbic structures changes		
Increased volume in limbic structures; especially pronounced in the cingulate and right inferior frontal gyrus	(*n* = 1) Madide et al. (2012)	
Increased volume bilaterally in limbic cortices of the anterior and posterior cingulate, ventral and medial temporal lobes, and bilateral perisylvian cortices	(*n* = 1) Sowell et al. (2010)	
Striatal and limbic structures increased vulnerability	(*n* = 1) Roussotte et al. (2010)	
Putamen changes		
Increased connectivity between the putamen seeds and frontal brain	(*n* = 1) Roussotte et al. (2012)	
Increase in left putamen volume	(*n* = 1) Roos et al. (2014)	
Negative correlation between performance and activation in: left parahippocampal gyrus, in the pre‐ and post‐central gyri bilaterally, and superior temporal gyrus and putamen	(*n* = 1) Roussotte et al. (2011)	
Putamen enlargement	(*n* = 1) Tsai et al. (2019)	
Reduction in subcortical region of the putamen; associated with delayed verbal memory and poor sustained attention	(*n* = 1) Madide et al. (2012)	
Striatum changes		
Damage in fronto‐striatal circuit; associated with decreased recruitment in working memory	(*n* = 1) Roussotte et al. (2011)	
Greater severity of damage in striatum; potentially associated with more severe cognitive deficits	(*n* = 1) Madide et al. (2012)	
Greater volume in striatal and associated regions (when comparing exposed males to male controls)	(*n* = 1) Roos et al. (2014)	
Reduced volume in striatal structures bilaterally, left parieto‐occipital and right anterior prefrontal cortices	(*n* = 1) Sowell et al. (2010)	
Severe volume reduction in striatum (in comparison to alcohol‐exposed group when compared with controls)	(*n* = 1) Sowell et al. (2010)	
Striatal volume reduction; linked to cognitive deficits	(*n* = 2) Madide et al. (2012); Tsai et al. (2019)	
Volume reduction in striatal region bilaterally	(*n* = 1) Madide et al. (2012)	
Thalamus changes		
Greater volume in the right ventral diencephalon (in exposed males when compared with exposed females)	(*n* = 1) Roos et al. (2014)	
Reduction in thalamus	(*n* = 1) Gutwinkski (2017)	
Reduced levels of myoinositol in thalamus; possibly linked to poor performance on visual motor integrations tasks	(*n* = 1) Smith (2017)	
Reduced recruitment of left and right thalamus during working memory versus rest	(*n* = 1) Roussotte et al. (2011)	
Volume reduction in thalamic regions bilaterally	(*n* = 1) Madide et al. (2012)	(*n* = 1) Warton, Taylor et al. (2018)

Abbreviations: HPA, hypothalamus‐pituitary‐adrenal; K‐CPT, Conners' Kiddie Continuous Performance Test; PME, prenatal methamphetamine exposure.

^a^ Articles referenced in this table are cited in full in Appendix S2: Annotations.

### 3.6 Interventions and recommendations

Search results relating to programs and recommendations were labelled as interventions. The search strategy resulted in the identification of seven programs that specifically support children with PME and/or mothers who used MA during pregnancy, which are summarized in Table 6. The programs identified offer support from the prenatal period through to postpartum, with some extending follow‐up supports until the infant's first birthday or beyond.^21^, ^22^ Many of the programs use an interdisciplinary team of providers to meet a range of needs for their clients including health, social and interpersonal needs that extend beyond conventional notions of perinatal health and substance use.^23^, ^24^, ^25^ For example, The University of Hawaii's Perinatal Addiction Treatment of Hawaii (PATH) Clinic offers a wide range of supports through self‐nurturing and craft‐based groups focusing on nutrition, self‐esteem, parenting skills, and relationships.^25^ Programs such as UNC Horizons Clinic and University of Hawaii's PATH Clinic are noteworthy for embracing women's self‐identified needs as opposed to predetermined supports.^23^, ^25^ Recommendations for further support measures identified in the results are presented in Table 7, and include family‐oriented and gender‐specific support, and a preference for in‐resident treatment over “outpatient” care.

**TABLE 6:** Interventions^a^

Program or intervention	Description	Mentioned In (source)
CTSA's Pregnancy and Opioids Models of Care (PROMO)	Program details unavailable, please contact Smid (2017) for any inquiries.	Smid (2017)
Nurse‐Family Partnership	A community health program that empowers vulnerable first‐time mothers via the support and development of relationships, between trained nurses and first‐time mothers. Through regular home visits from early pregnancy through to 24 months, women receive the support they need to engage in important prenatal care, parenting and responsible care, as well as, future planning for mother and baby (1). Mothers living with addiction during pregnancy are supported by nurses, who are able to connect them to relevant services while an interdisciplinary team of specialists including substance abuse counselors, obstetricians, pediatricians, social workers, and mental health specialists provide overall care (2). See more program details at: 1. https://www.nursefamilypartnership.org/wp‐content/uploads/2020/08/NFP‐Overview‐1.pdf 2. https://www.nursefamilypartnership.org/wp‐content/uploads/2020/03/NFP‐and‐Opioids_20201004‐1.pdf	Abar et al. (2014)
Project Nurture	Offers a variety of prenatal and postnatal support services for women living with complex life circumstances including addiction. This includes peer support, residential and in‐service treatment, medication assisted treatment with the use of buprenorphine and methadone, as well as level 1 outpatient addiction treatment. Mother and baby are followed for approximately 1 year postpartum. Operations are facilitated out of three sites located in the Portland Metropolitan area (USA). See more program details at: https://www.healthshareoregon.org/storage/app/media/documents/Commitment%20to%20Health/Project%20Nurture%20‐%20Learn%20More%20Flyer.pdf ^b^	Chatterjee (2018)
Substance Use in Pregnancy Recovery Addiction Dependence Clinic (SUPeRAD)	A speciality prenatal clinic, focused on providing holistic support to women with substance use disorders. Support services include, maternal fetal medicine, addiction specialists, medication‐assisted treatment with buprenorphine, recovery peer support, and resource management. See more program details at: https://medicine.utah.edu/internalmedicine/epidemiology/parcka/patient‐care/	Smid (2017)
UNC's Horizons Clinic	A women centered substance use disorder treatment program, offering interdisciplinary supports for pregnant women, parenting women, and/or those affected by abuse or violence (1). Uses an attachment (2) and trauma‐responsive model of care, to work towards healing of both mom and baby (1). A combination of medication and counseling services are offered for the treatment and recovery from substance use disorders (1). Programming integrates residential and outpatient services, prenatal and postpartum care, family planning (1), career counseling, housing assistance, case management, family therapy, and child development services to the children of the women they care for (2). See more program details at: 1. https://www.med.unc.edu/obgyn/horizons/ 2. https://www.med.unc.edu/obgyn/horizons/about‐us/who‐we‐are/	Smid (2017)
University of Hawaii's Perinatal Addiction Treatment of Hawaii (PATH) Clinic of Waikiki Health	The program provides a patient‐centered harm reduction approach, whereby clients are encouraged to participate in as many services they feel comfortable with. Supports comprise of prenatal and postpartum care, childcare, transportation, addiction psychiatry services (trauma informed counseling), case management, and assistance with housing. Group classes are offered, and include childbirth, parenting, smoking cessation, healthy eating, and prevention of relapse. Other group classes with the focus on strengthening women's self‐esteem and maternal‐infant bonding have also been implemented. These include self‐nurturing and craft‐based groups. Further, groups have been created to support the needs of the relationships between women and their partners. Due to a strong interest in providing healthy nutritional food to their clients, a healing garden has been developed, on site. This carefully planned garden offers an abundance of fruits and vegetables, which are provided to the women in the programme. Through processes of contingency management, they also offer motivational incentives for women. The hope is that these incentives may heighten resilience to which the women may overcome the barriers they experience in clinic participation. See more program details at: https://www‐ncbi‐nlm‐nih‐gov.cyber.usask.ca/pmc/articles/PMC3292917/	Smid (2017)
Women's Alcohol and Drug Service (WADS)	Provide multidisciplinary, specialist clinical services to pregnant women living with complex substance use dependence. Additionally, the program provides medical care to infants exposed prenatally to drugs and alcohol. See more program details at: https://www.thewomens.org.au/health‐professionals/maternity/womens‐alcohol‐and‐drug‐service	The Royal Women's Hospital (2017)

^a^ For full references and summaries of sources please see Appendix S2: Annotations.

^b^ All links to further information in this table were active as of July 2021.

**TABLE 7:** Recommendations^a^

Recommendations	Mentioned in (source) (*n*)
Care rooted in acceptance for MA use disorder as disease, with removal of punishment and stigmatization	(*n* = 1) Dinger et al. (2017)
Family‐oriented and gender‐specific approach to harm reduction for addiction in pregnancy	(*n* = 1) Smid (2017)
Greater parental monitoring and home life for children with PME	(*n* = 1) Smith et al. (2016)
Involvement with prenatal services such as monthly ultrasound can act as a strong motivator for addiction treatment	(*n* = 1) Chatterjee (2018)
Multidisciplinary interventions/approaches for mothers that use MA during pregnancy	(*n* = 1) Gutwinski et al. (2017)
Reinforcement‐based therapy	(*n* = 1) Forray et al. (2015)
Residential treatment; alternatively, outpatient treatment providing 3–5 visits/week for the first several weeks, and 2–3 visits/week after that for at least 90 days	(*n* = 1) ACOG Committee (2011)

Abbreviations: MA, methamphetamine; PME, prenatal methamphetamine exposure.

^a^ Articles referenced in this table are cited in full in Appendix S2: Annotations.

Additional outcomes of interest including long‐term outcomes, trimester‐related effects, dose‐response effects, and results specific to Indigenous people are collated and summarized below as they were distributed throughout the various categories.

### 3.7 Long‐term outcomes

Other outcomes, were scarce in the reviewed articles, probably because of the time frames of ongoing prospective studies. However, two suggestions for later life outcomes were identified: the first proposed that PME may be associated with an increased risk in males for drug‐induced neurotoxicity as adults.^26^ The other hypothesized that as a result of the link between fetal growth restriction and metabolic syndrome and obesity, children with PME prone to changes in fetal growth parameters may also be at risk for similar diseases.^27^

### 3.8 Trimester‐related effects

As a result of limits in the design methods, a large majority of the literature did not comment on effects related to trimester of MA exposure. Among trimester effects noted, Wright et al.^28^ reported an association between PME and children born with a positive meconium or urine toxicology at birth being smaller than those only exposed in the first trimester. LaGasse et al.^29^ found that first‐trimester exposure was associated with an increased total stress/abstinence and physiological stress, and third‐trimester exposure was associated with increased lethargy and hypotonicity. Finally, maternal weight gain was greater in those who stopped using MA in the first and second trimesters than in those who continued use into the third trimester.^30^

### 3.9 Dose‐response effects

Several outcomes were reported to be associated with more frequent drug use and/or higher amounts of MA, including an increased stress response in the neonatal period, poorer fine motor scores, and poorer inhibitory control^31^; increased response in cortisol reactivity^32^; higher scores for attention problems and being withdrawn^33^; lower grasp scores^34^; lower arousal and excitability^29^; greater fetal losses^35^; and increased anxiety, depression, and attention problems at ages 3 and 5 years.^26^

### 3.10 Indigenous‐specific results

Among the articles reviewed, Wouldes et al.,^36^ and Smid^26^ are the only ones to discuss findings specific to Indigenous people. Wouldes et al.^36^ reported evidence of delayed cognition and fine motor performance in Māori males during infancy and toddlerhood after PME, whereas Smid^26^ references the National Longitudinal Study of Adolescent Health, which reported higher rates of MA use among Native American youth compared with other ethnicities, but provided no further information on impacts or outcomes. Implications of these and general findings for Indigenous women are discussed below.

## 4 DISCUSSION

Prenatal methamphetamine exposure is increasingly a concern as the number of users, specifically those of childbearing age, continues to rise.^13^ Our findings indicate that MA use in pregnancy is associated with numerous consequences for mother and baby. Women who use MA may experience an increased risk for a variety of diagnoses, including hypertensive diseases and psychiatric disorders. Infants with PME may experience preterm birth, shorter gestation, impacts on anthropomorphic measures including decreased birth weight, and affective disorders in childhood.

Much of the literature on effects of PME in children stemmed from the Infant Development, Environment, and Lifestyle (IDEAL) study, a cross‐national, longitudinal prospective study on maternal and child outcomes following PME from birth to age 7.5 years.^31^ Some IDEAL reports attributed nation‐specific results to differences in government policies and support.^20^ For example, the USA enforces a legal mandate requiring health professionals to report drug use during pregnancy to Child Protective Services; such referrals are associated with inadequate prenatal care.^37^ The impacts of such policies on prenatal care and subsequent maternal and child health affect pregnant women who use MA,^38^ both in USA and in Canada. For instance, birth alerts are a policy used among many provinces for decades in which hospital staff notify Child Protective Services about a potential risk to an unborn child without the parents’ awareness.^39^ This process often results in infants being placed into government care once they are born (also known as “child apprehension”), which disproportionately affects Indigenous people and echos other acts of colonization such as Residential Schools and the 60s Scoop.^39^ Although some provinces have recognized the implications of and have since dismantled this policy, the associated practices continue.^39^

To facilitate care that minimizes the harmful effects of PME we need to “avoid stigmatizing labels that lead to punitive policies”.^40^ Our results articulate recommendations for the acceptance of MA use disorder as a disease that requires medical, psychological, and social supports.^38^ Interventions that embody this approach have the potential to minimize the effects of MA use in pregnancy for mothers and babies.

Although literature reviewed did not specifically focus on Indigenous women with MA use during pregnancy, Indigenous women and their offspring are likely subject to physiological and biological effects of PME identified in our review. However, based on our knowledge of health inequities stemming from colonial practices, including findings such as delayed cognitive and fine motor performance in Māori males with PME,^36^ there is a potential for poorer outcomes among Indigenous women and their infants. Strengths‐based approaches that center on traditional and cultural teachings show great promise in supporting Indigenous women and their children in their journeys with MA use and PME.^16^ The overall dearth of information on effects of PME and interventions for Indigenous women experiencing complex life circumstances and their offspring highlights a need for additional targeted research by and with Indigenous scholars to understand the particular impacts of MA use during pregnancy and PME within relevant historical and sociocultural contexts.^4^

It is important to note certain limitations inherent in our results. Specifically, it was not always clear if studies controlled for confounding variables, and often data for an outcome were limited to a small number of studies. In addition, results for many outcomes were not definitive. Finally, some studies may include a population not representative of the whole spectrum of those who use substances in pregnancy, as universal screening is not typical. These limitations point to the need for additional well‐defined and controlled studies on impacts and mediating factors of MA use in pregnancy.

Future research should broaden this scan to identify additional interventions and strategies for addressing MA use in pregnancy, particularly those developed by, with, and for Indigenous women. Our results did not include publications on effective interventions for use of other substances in pregnancy, which may also hold promise for addressing PME. In addition, including literature published before 2010 would likely capture other interventions, including work on early models of wrap‐around care for Indigenous women with substance‐use disorders.^4^ Finally, evaluation studies comparing outcomes for women and babies involved in innovative harm reduction interventions with standard care could provide further evidence towards best practices in mitigating effects of PME and MA use in pregnancy.

The present review reasserts that interventions must address the unique and intersecting contexts of Indigenous women and their families.^1^, ^3^, ^4^, ^7^ Integrated programs such as Sanctum 1.5 that include prenatal care, parenting supports, and the facilitation of socioeconomic supports and services in a culturally safe manner are necessary and promising for Indigenous women and their offspring impacted by MA use in pregnancy.^1^, ^4^, ^7^ The present review can inform promising and effective interventions for Indigenous women who use MA during pregnancy and their children that address social and cultural needs alongside health needs, which may positively impact individuals, communities, and future generations.

## CONFLICTS OF INTEREST

The authors have no conflicts of interest.

## AUTHOR CONTRIBUTIONS

MA and CM contributed to the design of the work, data collection, analysis, and interpretation of data, and drafted and revised the article. LE contributed to the analysis and interpretation of data, and drafted and revised the article. KG contributed to the design of the work, analysis, and interpretation of data, and drafted and revised the article. AK contributed to the design of the work, data collection, analysis, and interpretation of data, and drafted and revised the article. All authors have approved the final version and agree to be accountable for all aspects of this work and ensure that questions related to the accuracy and integrity of any part of the work are appropriately investigated and resolved.

## Supporting information

Appendix S1

Appendix S2
