# Supplementary material for: An environmental scan of impacts and interventions for women with methamphetamine use in pregnancy and their children
Source: Int J Gynaecol Obstet. 2021 Aug 23;155(2):220–38. doi: 10.1002/ijgo.13851 (PMC9291965; doi:10.1002/ijgo.13851)
Supplement: Supplementary file 1 — Appendix S1 [file IJGO-155-220-s002.pdf]

Search Strategies

Initial search criteria: (1) English language, (2) research with human participants, (3) date range January 1, 2005 to January 1, 2020.

Search Strategy for Academic Literature:

Medline

1. Fetus/
2. exp Pregnancy/
3. Pregnancy Complications/
4. Pregnant Women/
5. Maternal Exposure/
6. Prenatal Exposure Delayed Effects/
7. or/1-6
8. ((pregnan* or prenat* or pre-nat* or perinat* or antenatal or matern* or mother?) adj5 (expos* or abus* or "use" or use* or consum* or dependance)).mp.
9. (expos* adj5 (fetus* or fetal or newborn? or neonat* or baby or babies or birth or "in the womb" or "in utero")).mp.
10. or/8-9
11. exp Infant/
12. Child, Preschool/
13. Child/
14. Mothers/
15. or/11-14
16. pregnan*.mp.

17. prenat*.mp.

18. pre-nat*.mp.

19. or/16-18

20. 15 and 19

21. Methamphetamine/

22. methamphetamine?.mp.

23. crystal meth.mp.

24. or/21-23

25. 7 or 10 or 20

26. 24 and 25

27. limit 26 to (english language and humans and yr="2005 - 2020")

28. remove duplicates from 27

Embase

1. fetus/

2. exp pregnancy/

3. pregnancy complication/

4. pregnant woman/

5. maternal exposure/

6. prenatal exposure/

7. or/1-6

8. ((pregnan* or prenat* or pre-nat* or perinat* or antenatal or matern* or mother?) adj5 (expos* or abus* or "use" or use* or consum* or dependance)).mp.

9. (expos* adj5 (fetus* or fetal or newborn? or neonat* or baby or babies or birth or "in the womb" or "in utero")).mp.

10. or/8-9

11. exp infant/

12. preschool child/

13. child/

14. mother/

15. or/11-14

16. pregnan*.mp.

17. prenat*.mp.

18. pre-nat*.mp.

19. or/16-18

20. 15 and 19

21. methamphetamine/

22. methamphetamine?.mp.

23. crystal meth.mp.

24. or/21-23

25. 7 or 10 or 20

26. 24 and 25

27. limit 26 to (human and english language and exclude medline journals and yr="2005 - 2020")

28. remove duplicates from 27

CINAHL

S1	(MH "Fetus")
S2	(MH "Pregnancy+")
S3	(MH "Pregnancy Complications")
S4	(MH "Expectant Mothers")
S5	(MH "Maternal Exposure")
S6	(MH "Prenatal Exposure Delayed Effects")
S7	S1 OR S2 OR S3 OR S4 OR S5 OR S6
S8	pregnan*
S9	prenat*
S10	pre-nat*
S11	perinat*
S12	antenatal
S13	matern*
S14	mother
S15	S8 OR S9 OR S10 OR S11 OR S12 OR S13 OR S14
S16	expos*
S17	abus*
S18	use""
S19	use*
S20	consum*
S21	dependance
S22	S16 OR S17 OR S18 OR S19 OR S20 OR S21

S23 S15 AND S22

S24 fetus

S25 fetal

S26 newborn

S27 neonat*

S28 baby

S29 babies

S30 birth

S31 in the womb""

S32 in utero""

S33 S24 OR S25 OR S26 OR S27 OR S28 OR S29 OR S30 OR S31 OR S32

S34 S16 AND S33

S35 S23 OR S34

S36 (MH "Infant+")

S37 (MH "Child, Preschool")

S38 (MH "Child")

S39 (MH "Mothers")

S40 S36 OR S37 OR S38 OR S39

S41 S8 OR S9 OR S10

S42 S40 AND S41

S43 (MH "Methamphetamine")

S44 methamphetamine

S45 crystal meth

S46 S43 OR S44 OR S45

S47 S7 OR S35 OR S42

S48 S46 AND S47

S49 S46 AND S47*

ProQuest

((MESH.EXACT("Methamphetamine") OR methamphetamine? OR (crystal meth)) AND ((MESH.EXACT.EXPLODE("Fetus") OR MESH.EXACT.EXPLODE("Pregnancy") OR MESH.EXACT("Pregnancy Complications") OR MESH.EXACT("Pregnant Women") OR MESH.EXACT("Maternal Exposure") OR MESH.EXACT("Prenatal Exposure Delayed Effects")) OR (((pregnan* OR prenat* OR pre-nat* OR perinat* OR antenatal OR matern* OR mother?) NEAR/5 (expos* OR abus* OR "use" OR use* OR consum* OR dependance)) OR (expos* NEAR/5 (fetus* OR fetal OR newborn? OR neonat* OR baby OR babies OR birth OR "in the womb" OR "in utero"))) OR ((MESH.EXACT.EXPLODE("Infant") OR MESH.EXACT("Child, Preschool") OR MESH.EXACT("Child") OR MESH.EXACT("Mothers")) AND (pregnan* OR prenat* OR pre-nat*)))) AND human(yes) AND la.exact("English")

<u>Search Strategy for Google:</u>

Methamphetamine Pregnancy Approach
