# Supplementary material for: An environmental scan of impacts and interventions for women with methamphetamine use in pregnancy and their children
Source: Int J Gynaecol Obstet. 2021 Aug 23;155(2):220–38. doi: 10.1002/ijgo.13851 (PMC9291965; doi:10.1002/ijgo.13851)
Supplement: Supplementary file 2 — Appendix S2 [file IJGO-155-220-s001.pdf]

**Citation**<br>Annotation	**Output Codes[1]**
**Abar B, LaGasse LL, Derauf C, Newman E, Shah R, Smith LM, Arria A, Huestis M, Della Grotta S, Dansereau LM, Neal C, Lester, BM. Examining the relationships between prenatal methamphetamine exposure, early adversity, and child neurobehavioral disinhibition. *Psychol Addict Behav.* 2013;27(3):662–673. DOI: 10.1037/a0030157**<br>This study investigated whether children who experienced prenatal methamphetamine exposure (PME) exhibited neurobehavioral disinhibition, and if early adversity in the first three years of life mediated this relationship. Neurobehavioral disinhibition (ND) was assessed using a two-factor model which examined: a) behavioral control, and b) executive function. The authors reported on a similar study done by Tarter et al. (2003), which studied ND using three dimensions: behavioral control, emotional regulation and executive cognitive functioning. The current study reported that children with PME presented significantly more emotionally reactive behavior, anxiety and/or depression, and aggression at five years of age than comparison children. The study suggested an association between PME and a child's behavioral and emotional control at five years of age, which was further associated with executive function deficits at 6.5 years of age. Additionally, they found early adversity had a significant mediating role between PME and ND. They mentioned efforts by LaGasse et al. (2011) which reported that cross-cultural postnatal service provisions might reveal a protective influence of postnatal services on child development post-PME. They also commended the Nurse - Family partnership (Olds, 2006) as a potential service to be replicated in efforts to minimize the effects of PME.	B<br>C
**Abar B, LaGasse LL, Wouldes T, Derauf C, Newman E, Shah R, Smith LM, Arria AM, Huestis MA, Della Grotta S, Dansereau LM, Wilcox T, Neal CR, Lester BM. Cross-national comparison of prenatal methamphetamine exposure on infant and early child physical growth: a natural experiment. *Prev Sci.* 2014;15(5):767–776. DOI: 10.1007/s11121-013-0431-5**<br>This study used cross-national data from the IDEAL study's United States (US) and New Zealand (NZ) cohorts to examine the effects of PME and country of origin on length/height and weight. They reported that the negative influence of PME on length/height was greater for those in the US than in NZ. However, weight was not affected by country of origin: children with PME in both cohorts demonstrated shorter and lighter measurements when compared to their respective controls. The authors suggested that the height difference between nations was likely due to government policy and differences in care provided to mothers and their infants with PME. They referenced Wu et al. (2012) who reported higher rates of inadequate prenatal care in the US when compared to NZ. According to Abar et al, Wu's findings were likely a result of NZ's policies around free health care and lack of fear of child apprehension due to a lack of mandatory reporting laws. However, the article by Wu et al. (2012) stated that statistical tests showed no difference in insurance coverage the US between children with PME and their comparisons. Abar et al. (2014) recommended programs like Nurse-Family Partnership to prevent the influences of inadequate pre- and post-natal care.	I<br>N/I

**American College of Obstetricians and Gynecologists (ACOG) Committee on Health Care for Underserved Women. Committee Opinion No. 479: Methamphetamine abuse in women of reproductive age. *Obstet Gynecol.* 2011;117(3):751–755. DOI: 10.1097/AOG.0b013e318214784e**<br>The article synthesized literature that found PME was associated with lower birth weights and increased rates of infants presenting as small for gestational age (SGA). Although the authors acknowledged the inadequate controls used in a Swedish study, they referenced other studies with matched controls having similar results. An ongoing prospective study which found that PME was associated with decreased arousal, increased stress and poor quality of movement in the newborns was also mentioned. Another study reported lower scores on tests of attention, visual motor integration, verbal memory and long-term spatial memory in teens aged 13 to 16, with PME. They reported that imaging studies such as MRI studies have revealed a reduced volume in the putamen, globus pallidus and hippocampus associated with poorer performance on attention and memory tasks. In review of fMRI studies, brain activation during verbal memory tasks in children with PME showed more diffuse patterns than those without. One study of 276 pregnant women reported lower Apgar scores at one minute and five minutes, and higher neonatal mortality and maternal intensive care unit admissions among children with PME. The article further mentioned they found no literature supporting an increase in birth defects among children with PME. They concluded that women that use methamphetamine (MA) during pregnancy are recommended to voluntarily seek residential treatment when possible. Alternatively, outpatient treatment should be provided with three to five visits per week for the first several weeks, and two to three per week after that to provide at least 90 days of support after initiation.	B<br>C<br>I<br>N<br>N/I
**Behnke M, Smith VC, Committee on Substance Abuse, Committee on Fetus and Newborn. Prenatal substance abuse: short- and long-term effects on the exposed fetus. *Pediatrics.* 2013;131(3):e1009–e1024. DOI: 10.1542/peds.2012-3931**<br>This review discussed the prevalence, ability to identify exposure, effects in newborn period and long-term effects of various drugs used during pregnancy. The discussion regarding outcomes for prenatal methamphetamine use was limited, but they brought attention to literature that documented changes in growth and infant neurobehavior. More specifically, they mentioned results from the IDEAL study which have shown changes in neurobehavior presenting as poor movement quality, decreased arousal and increased stress. They referenced Smith et al.'s (2003) documentation of withdrawal symptoms but disregarded its significance due to the small need for pharmacological intervention among those infants. Lastly, they found no literature had yet associated PME with congenital anomalies or long-term effects.	B<br>N/I
**Brecht M-L, Herbeck DM. (2014). Pregnancy and fetal loss reported by methamphetamine-using women. *J Subst Abuse Treat.* 2014;8, 25-33. DOI: 10.4137/SART.S14125**	M<br>N/I

They reported that a higher number of fetal losses were associated with increased severity of drug use, whereas lower drug use was associated with more live births. Overall, they reported that MA use in pregnancy resulted in a higher number of pregnancies with late-term fetal loss. They also observed a higher number of terminated pregnancies without a live birth.	
**Brinker MJ, Cohen JG, Sharrette JA, Hall TA. Neurocognitive and neurodevelopmental impact of prenatal methamphetamine exposure: A comparison study of prenatally exposed children with nonexposed ADHD peers.** ***Appl Neuropsychol.*** **2019;8(2):132–139. DOI: 10.1080/21622965.2017.1401479**<br>This study explored the neurocognitive and neurodevelopment effects of using MA during pregnancy. IQ scores and Wechsler Intelligence Scale, Fourth Edition (WISC-IV) were compared between children with PME and clinically-referred children diagnosed with ADHD. The groups were not matched on age or gender but had similar demographics. Results showed that children who were exposed to MA had a significant decrease in scores when compared to children with ADHD. Further analysis accounting for alcohol use showed a similar result for all scores except for Processing Speed Index scores. Results also suggested a potential reduction in processing speed as children with PME age. Regression analysis indicated gender was not a significant predictor for the WISC-IV scores.	C
**Burger A, Roos A, Kwiatkowski M, Stein DJ, Donald KA, Howells FM. The effect of in utero exposure to methamphetamine on brain metabolism in childhood using 1H-magnetic resonance spectroscopy (1H-MRS).** ***S Afr J Psychiatry.*** **2015;21(3):109. [no DOI]**<br>Their recent study found changes in cortical thickness and brain volume in six-year-olds with PME. The study focused on investigating changes in neurometabolites in the anterior cingulate cortex and dorsolateral prefrontal cortex in children with PME. Using a single voxel proton spectroscopy (^{1}H-MRS), the researchers reported the standard ^{1}H-MRS metabolite concentrations for children with PME and children without PME. As this was a presentation abstract, results were not yet reported.	N
**Canadian Centre on Substance Use and Addiction. Methamphetamine [CCSA website]. 2018. https://www.ccsa.ca/sites/default/files/2019-04/CCSA-Canadian-Drug-Summary-Methamphetamine-2018-en.pdf Accessed June 24, 2020.**<br>The document referenced literature that found methamphetamine use during pregnancy increased the risk for premature birth, low birth weight and abnormalities in the fetus's heart and brain.	N/I
**Cardwell MS. Late trimester hydranencephaly associated with maternal exposure to methamphetamine.** ***J Subst Use.*** **2014;19(5):351–352. DOI: 10.3109/14659891.2013.820801**<br>A 28-week ultrasound revealed normal anatomy, but fetal weight was borderline small for gestational age SGA. Four weeks later, a second ultrasound showed a fetus that was SGA and had an abnormal buildup of cerebrospinal fluid in the brain (hydrocephaly). Further examination allowed	N/I

for a diagnosis of hydranencephaly and SGA. The patient denied prior use of vasoconstrictive agents or other illicit drugs prior to the third trimester but admitted to heavy use of MA in the prior month. Urine toxicology screened positive for MA and cannabinoids. Evaluation was done for infectious agents associated with fetal intracranial abnormalities, but results presented negative. Readers must be mindful that hydranencephaly has been associated with causes of infections, bleeding diatheses, neoplasms and exposure to vasoconstrictive agents.	
**Chakraborty A, Anstice NS, Jacobs RJ, LaGasse LL, Lester BM, Wouldes TA, Thompson B. Prenatal exposure to recreational drugs affects global motion perception in preschool children.** ***Sci Rep.*** **2015;*5*(1). https://www.nature.com/articles/srep16921**<br>This study utilized the IDEAL study cohort to assess the impacts of PME on the development of visual brain areas. Global motion perception, a behavioral measure of visual development, was assessed in 145 children who were 4.5 years old. The results showed that prenatal exposure to alcohol or marijuana influenced visual development, whereas nicotine and MA did not. MA showed no effect on habitual visual acuity, stereopsis or global motion perception. This study showed that visual development can be influenced by prenatal drug exposure, however this effect was drug-specific. The low socioeconomic status of the entire group may have influenced the results.	C
**Chamberlain R. (2015). Methamphetamine and children.** ***Kai Tiaki: Nurs N Z.*** **2015;21(2):30–31. [no DOI]**<br>They reference literature which associated prenatal complications such as premature birth, altered neonatal behavior, abnormal reflexes, extreme irritability and congenital deformities with PME. They referred to Trecia Wouldes from Auckland University for further published outcomes related to PME.	B<br>N/I
**Chang L, Oishi K, Skranes J, Buchthal S, Cunningham E, Yamakawa R, Hayama S, Jiang CS, Alicata D, Hernandez A, Cloak C, Wright T, Ernst T. Sex-specific alterations of white matter developmental trajectories in infants with prenatal exposure to methamphetamine and tobacco.** ***JAMA Psychiatry.*** **2016;73(12):1217–1227. DOI: 10.1001/jamapsychiatry.2016.2794**<br>Mothers from the MA-and-tobacco exposed group were reported as having gained more weight during pregnancy than women in both the tobacco exposed and unexposed groups. Diffusion tensor imaging (DTI) identified that boys exposed to MA/tobacco showed lower fractional anisotropy (FA) and higher diffusivity measures in the superior corona radiata (SCR) and mean and radial diffusivity (RD) in the posterior corona radiata (PCR) which eventually normalized at the later age of three months. These differences in trajectories were not observed in the female groups, but FA was lower in the anterior corona radiata of the females exposed to stimulants. Female groups exposed to stimulants also tended to show slower development through radial ACR diffusivity than females who were unexposed. For both stimulant groups, the retrolenticular internal capsule demonstrated an age-dependent decrease in axial diffusivity when compared to infants who were not exposed. The	M<br>N/I

authors reported that infants exposed to amphetamine/tobacco had delayed development on active muscle tone and total Amiel - Tison Neurological Assessment at Term (ATNAT) scores, but that these measures normalized by the age of three to four months. They suggested normalization of motor examination and SCR and PCR trajectories might be associated with improved myelination following cessation of prenatal exposure.	
**Chatterjee, R. Another drug crisis: Methamphetamine use by pregnant women [NPR website]. 2018. https://www.npr.org/sections/health-shots/2018/11/29/668487475/another-drug-crisis-methamphetamine-use-by-pregnant-women Accessed June 24, 2020.**<br>They referenced Dr. Lindsay Admon, an assistant professor of obstetrics and gynecology at the University of Michigan, who shared that long term MA use in pregnancy increased the risk of women dying during or after childbirth. There was also the possibility of acquiring lifelong health complications such as the need for a blood transfusion, heart failure, cardiac arrest and eclampsia. They also reported a further possibility for maternal death that was identified by Dr. Tricia Wright, an associate professor at the University of Hawaii. A current study was referenced, which reported an increased risk of premature birth and placental abruption. They identified a program called Project Nurture, that provides behavioral therapy and residential treatment to support pregnant women with substance use disorders. They interviewed a mother who sought assistance and support in Colorado, US during her pregnancy for the use of MA. This woman shared that monthly ultrasounds were her greatest motivator for her treatment.	I<br>M<br>N/I
**Colby JB, Smith L, O'Connor MJ, Bookheimer SY, Van Horn JD, Sowell ER. White matter microstructural alterations in children with prenatal methamphetamine/polydrug exposure. *Psychiatry Res.* 2012;204(2-3):140–148. DOI: 10.1016/j.pscychresns.2012.04.017**<br>This study examined changes in white matter microstructure in children with PME. In effort to reflect real life populations, three groups of children were investigated: a) children with PME (also exposed to alcohol) b) children with prenatal alcohol exposure but no PME and c) typically developing children. DTI and neurocognitive testing revealed children exposed to MA demonstrated higher fractional anisotropy (FA) mainly in the left-sided regions, including a large portion of the left anterior corona radiata (LCR). They suggested this diffusion pattern was likely a result of lower radial diffusivity (RD) moreso than higher axial diffusivity (AD). To explain the clinical relevance of these findings, tests of executive functioning and visuomotor integration were performed. This revealed FA in the right external capsule was associated with performance on a test of visuomotor integration across each group.	C
**Derauf C, Lagasse LL, Smith LM, Newman E, Shah R, Neal CR, Arria AM, Huestis MA, Dellagrotta S, Dansereau LM, Lin H, Lester BM. Prenatal methamphetamine exposure and inhibitory control among young school-age children. *J Pediatr.* 2012;161(3):452–459. DOI: 10.1016/j.jpeds.2012.02.002**<br>This study explores inhibition control and working memory in children with PME. The IDEAL study measured these characteristics using the Hearts	B

and Flowers version of the Dots task from the Directional Stroop Battery for school age children at a 66-month/5.5-year-old follow-up. The authors suggested that the tests for working memory present a stronger effect on performance as opposed to memory in young children. They described inhibitory control as a factor of executive function relating to emotional and cognitive control. They found heavy exposure was associated with reduced accuracy in both incongruent and mixed conditions on the Stroop-like task. Caregiver psychological distress and child mistreatment were also associated with reduced accuracy in the incongruent condition. Caregiver psychological symptoms were further associated with reduced accuracy in mixed conditions and a longer reaction time in the congruent condition, but decreased reaction time in the mixed. Their findings indicated a reduction in inhibitory control among children with PME.	
**Derauf C, Lester BM, Neyzi N, Kekatpure M, Gracia L, Davis J, Kallianpur K, Efird JT, Kosofsky B. Subcortical and cortical structural central nervous system changes and attention processing deficits in preschool-aged children with prenatal methamphetamine and tobacco exposure. *Dev Neurosci.* 2012;34(4):327–341. DOI: 10.1159/000341119**<br>Automated Segmentation was used to measure subcortical and cortical structural volumes and cortical thickness to assess for changes in three- to five-year-old children with PME. Attention was also assessed in this population using Conners' Kiddie Continuous Performance Test Version 5. Through these methods, the authors found that PME was associated with significant volume reductions in the caudate nucleus of preschool-aged children. They also found PME-associated cortical thickness increases in perisylvian and orbital-frontal cortices. Their findings associated PME to a worse reaction time on Conners' Kiddie Continuous Performance Test (Version 5) by inter-stimulus interval, a measure defined as the ability to adjust to changing task demands. Caudate volume was inversely related to this reaction time. With these relationships in mind, the authors suggested that children with PME may have attentional deficits mediated by caudate volume reductions.	B<br>N
**Diaz SD, Smith LM, LaGasse LL, Derauf C, Newman E, Shah R, Arria A, Huestis MA, Della Grotta S, Dansereau LM, Neal C, Lester BM. Effects of prenatal methamphetamine exposure on behavioral and cognitive findings at 7.5 years of age. *J Pediatr.* 2014;164(6):1333–1338. DOI: 10.1016/j.jpeds.2014.01.053**<br>This study examined the effects of PME on cognitive and behavioral outcomes at 7.5 years of age. The children were initially recruited for the IDEAL study and those who followed up at 7.5 years were included. Behavior and cognition were assessed using the Conners' Parent Rating Scale-Revised: Short Form. Through this assessment, children with PME appeared to have higher cognitive problems subscale scores and were also more likely to have above average cognitive problems scores. On the other hand, behavioral problems measured by oppositional, hyperactivity and attention-deficit/hyperactivity disorder index subscales showed no association to PME. In previous IDEAL findings, children of five years of age with PME demonstrated externalizing and ADHD problems. Those results were attained by child-based measures as opposed to the parent reports used in this study.	B<br>C

**Dinger J, Hinner P, Reichert J, Rüdiger M. Methamphetamine consumption during pregnancy – effects on child health. *Pharmacopsychiatry.* 2017;50(3):107–113. DOI: 10.1055/s-0042-122711**<br>The authors found that pregnant women using MA were at a greater risk for developing preeclampsia and gestational hypertension than women who did not use MA. They also associated an increased likelihood for fetal demise, abortion or preterm delivery as complications linked to MA use during pregnancy. They add that infants with PME were more likely to be born with lower birth weights than expected for gestational age. In addition, odds for neonatal or infant death were significantly heightened in infants with PME. They discussed teratogenic findings suggesting alterations in brain structures and congenital malformations due to MA use in pregnancy. However, due to the scarcity of case-control and prospective studies confirming these findings, they cited others who suggested PME teratogenicity was unlikely. They described newborn withdrawal from PME including presentation with more lethargic and poor movement quality, decreased arousal and increased stress during the first week of life in comparison to the withdrawal observed in infants exposed to opioids in pregnancy. Additionally, newborns with PME were described as more likely to exhibit poor sucking, jitteriness and excessive sucking. Infants with PME were also more likely to have smaller head circumference and were more prone to needing neonatal intensive care. They stated that the most prominent long-term effect of PME was on the development of the brain. Neuroimaging studies in children with PME showed volume reductions in the cortical and subcortical brain structures. The hypothalamic-pituitary-adrenal axis has been associated to changes caused by PME. Based on previous literature, they reported that at age three, children with PME had poorer motor performance; at age four: lower IQ scales; at age seven and a half: an increased likelihood of cognitive problems; at age eight: more problems with peers and aggressive behaviors; and by age fourteen, decreased school performance, specifically related to math, language and physical fitness as compared to controls. They discussed the influence of family background, parental stress and psychological symptoms experienced by primary caregivers on long-term development in children with PME. They also reported a lack of data on the effects of PME on the cardiovascular system. They acknowledged studies which state that resilience was lost in humans that used MA, along with epigenetic changes in the brain. They suggested transgenerational effects can be assumed. A fetus with PME may not only experience a lack of resilience, but also may be exposed to stresses in the home environment characterized by parents who use MA. The authors recommended that care for this population needs to be rooted in a theory of acceptance for MA-use disorder as a disease that requires medical, psychological and social support. They added that previous punitive interventions for "crack-babies" and their mothers were extremely stigmatizing and harmful and must be avoided in new care measures for PME.	B<br>C<br>I<br>M<br>N<br>N/I
**Dyk JV, Ramanjam V, Church P, Koren G, Donald K. Maternal methamphetamine use in pregnancy and long-term neurodevelopmental and behavioral deficits in children. *J Popul Ther Clin Pharmacol.* 2014;21(2):e186–e196. https://jptcp.com/index.php/jptcp/article/view/329**	B<br>C

Griffiths Mental Developmental Scales (GMDS) revealed children with PME scored significantly lower on general performance, and at an age equivalent lower than their own age, in comparison to children without PME. Children with PME also scored lower on final General Quotients. Further differences were observed on the Personal and Social subscale, and Eye and Hand Coordination subscale. The Child Behavior Checklist (CBCL) revealed a tendency for children with PME to present with more atypical behaviors such as anxious/depressed behavior, somatic complaints, attention problems, aggressive behavior, affective problems, pervasive developmental problems, attention deficit/hyperactivity, oppositional defiant problems and sleep problems. Scores on both Aggressive Behavior and Attention Deficit/Hyperactivity Problems subscales presented a trend towards significance, but ultimately did not reach statistical significance.	
**Eze N, Smith LM, LaGasse LL, Derauf C, Newman E, Arria A, Huestis MA, Della Grotta SA, Dansereau LM, Neal C, Lester BM. School-aged outcomes following prenatal methamphetamine exposure: 7.5-year follow-up from the Infant Development, Environment, and Lifestyle Study.** ***J Pediatr.*** **2016;170:34–38.e1. DOI: 10.1016/j.jpeds.2015.11.070**<br>This was a 7.5-year, longitudinal, multicentre study (four sites) follow-up study to the IDEAL study. 209 children with completed Child Behavior Checklist Data were included. The study determined if early adversity mediated the association between PME and behavioral problems. Adversity index scores were calculated using data from the first three years of life. The results showed that PME was associated with high adversity index scores, externalization, rule-breaking behavior and aggressive behavior. Indirectly, adversity demonstrated a mediating role for externalization. This study was longitudinal rather than cross-sectional.	B
**Forray A, Foster D. Substance use in the perinatal period.** ***Curr Psychiatry Rep*****, 2015;17(11):91. DOI: 10.1007/s11920-015-0626-5**<br>This review set out to summarize the current literature regarding prenatal substance use, the effects of its use on mother and infant and possible interventions for women using substances during pregnancy. The review individually discussed these items as they pertained to each substance. They associated PME with multiple outcomes: shorter gestational ages, lower birth weight, fetal loss, developmental and behavioral deficits, gestational hypertension, preeclampsia and intrauterine fetal death. They also drew attention to the outcomes' dependence on exposure and timing. To add to this, they referred to evidence of infants being smaller when exposed to MA in the first trimester only, as compared to infants without PME. They discussed interest in reinforcement-based therapy as an intervention for pregnant women using MA; however study results showed no difference between this intervention and control conditions.	B<br>I<br>M<br>N/I
**Galland BC, Mitchell EA, Thompson JMD, Wouldes T, NZ IDEAL Study Group. Auditory evoked arousal responses of 3-month-old infants exposed to methamphetamine in utero: A nap study.** ***Acta Paediatr.*** **2013;102(4):424–430. DOI: 10.1111/apa.12136**	N/I

The researchers aim was to determine if infants with PME have impaired arousal responses from sleep. Utilizing polygraphic nap studies, three-month-old children were studied during rapid eye movement (REM) and non-rapid eye movement (NREM) sleep. The authors reported no differences in arousal and sleep between PME and comparison groups. They were therefore unable to offer the reasoning that arousal deficits contributed to this population's increased susceptibility to sudden infant death syndrome.	
**Gargari SS, Fallahian M, Haghighi L, Hosseinnezhad-Yazdi M, Dashti E, Dolan K. Maternal and neonatal complications of substance abuse in Iranian pregnant women. *Acta Med Iranica.* 2012;50(6):411–416. https://pubmed.ncbi.nlm.nih.gov/22837120/** Neonatal abstinence syndrome was not reported in any of the MA users. Although the exposure group included a variety of drug exposures, the data showed MA use in pregnancy was linked to an increase in preterm births, younger gestational age, younger gestational age in preterm births, increased likelihood for NICU admission and increased likelihood for spending more days in the NICU. Mothers who used MA demonstrated an increased likelihood for anemia and post-partum hemorrhages. Infants with PME also were observed to have a lower birth weight, head circumference and smaller body length.	M<br>N/I
**Goldberg LR, Heiss CJ, White L, Kaf WA, Becker A, Schindler JB, Dion N, Oswalt J. (2010). Methamphetamine exposure, iron deficiency, and implications for cognitive-communicative function: A case study. *Communication Disorders Quarterly. 31*(3), 183-192. DOI: 10.1177/1525740109340437** The authors described results of an interdisciplinary evaluation of a four-year-and-eleven-month-old girl named Ann. Ann had diagnoses of congenital nystagmus, pale retinae (ocular albinism), right thalamic infarct in utero with left hemiparesis including the face, associated cerebral palsy, complex partial epilepsy and attention-deficit/hyperactivity disorder with features of opposition defiant disorder, and developmental delay. Medications included Tegretol for seizure disorder, Metadate for ADHD and a multivitamin. Parental grandmother reported "weak" crying and thrush shortly after vaginal birth. The mother was reported to have used MA, marijuana (daily) and tobacco during pregnancy. Ann was bottle-fed with no reports of difficulty in sucking and swallowing but had some difficultly transitioning to solid food. Early development included recurrent middle ear infections, chronic seizures and other environmental stress. Overall, they acknowledged Ann's difficulties in cognitive and communicative development could be associated with her diagnosis of ADHD, hypoxia during seizures or chronic iron deficiency.	B<br>C<br>N/I
**Good MM, Solt I, Acuna JG, Rotmensch S, Kim MJ. Methamphetamine use during pregnancy: Maternal and neonatal implications. *Obstet Gynecol.* 2010;116(2):330–334. DOI: 10.1097/AOG.0b013e3181e67094** This study found a significant increase in preterm births likely associated with lower initial Apgar scoring in children with PME. They also commented on an increased incidence of neonatal mortality among the exposed group as compared to the control group.	N/I

**Gorman MC, Orme KS, Nguyen NT, Kent EJ 3rd, Caughey AB. Outcomes in pregnancies complicated by methamphetamine use. *Am J Obstet Gynecol.* 2014;211(4):429.e1–7. DOI: 10.1016/j.ajog.2014.06.005**<br>This retrospective study aimed to characterize the maternal and neonatal outcomes associated with prenatal methamphetamine use. The International Classification of Diseases, (ICD-9), provided data for 8,542 women who identified using MA during pregnancy. The women were then compared with 2,031,328 control pregnancies. Their results suggested that mothers who used MA had an increased incidence of hypertensive diseases, frequency of pregnancy-associated hypertension, preeclampsia, severe preeclampsia and eclampsia. MA use was associated with preterm birth, abruption, intrauterine fetal death and infant death for those born at term. Babies born at term also had an increased incidence of neonatal death among MA using mothers, but after accounting for gestational age at delivery, this did not remain significant. SGA and low birth weight were also higher in children with PME. MA use also appeared to be protective against macrosomia and gestational diabetes mellitus. However, the use of ICD-9 limited the authors' ability to quantify the amount, frequency and timing of use.	M<br>N/I
**Gregory GA, Alton NR. Intrauterine illicit drug exposure and neurodevelopmental outcomes for children: How current literature informs management and assessment. *Pediatr Child Health.* 2017;27(8):383–387. DOI: 10.1016/j.paed.2017.05.003**<br>They reported on current studies that reported MA use in pregnancy was associated with smaller babies and accompanied by a shorter gestational period.	N/I
**Gutwinski S, Heinz A. Consumption of methamphetamine during pregnancy – transgenerational substance misuse? *Pharmacopsychiatry.* 2017;50(3):114–115. DOI: 10.1055/s-0043-102185**<br>The study mentioned Sowell et al.'s (2010) identification of volume reductions in brain regions such as the striatum and the thalamic and prefrontal lobe in children with PME. They also referenced Dinger at al.'s (2017) findings of poor motor function at three years old, lower IQ scores at four years old, and lower school performances in math and language in fourteen-year-old children with PME. They recommended that further investigation was needed to assess if PME increased the likelihood of developing a substance use disorder later in life. They acknowledged a collective interest among the authors they reviewed for multidisciplinary interventions/approaches for mothers who use substances during pregnancy.	C<br>I<br>N
**Hayward AR, DePanfilis D, Woodruff K. Parental methamphetamine use and implications for child welfare intervention: A review of the literature. *J Public Child Welf.* 2010;4(1):25–60. DOI: 10.1080/15548730903563095**<br>This paper reference IDEAL study findings which reported lower birth weight, smaller gestational size and neurobehavioral differences in infants with PME as compared to infants with no exposure.	B<br>N/I

**Heiss CJ, Goldberg LR, Dion N. Iron deficiency and overweight in a child exposed to methamphetamine in utero.** ***Top Clin Nutr.*** **2010;25(2):160–164. DOI: 10.1016/j.jada.2008.06.397**<br>They report use of the Food Processor computer diet analysis program to determine a three-day average nutrient intake for a child with PME. Analyses revealed adequate consumption of kilocalorie, protein, calcium, iron and major food groups. Her mother was reported to have used methamphetamine, marijuana and tobacco during pregnancy. The amount and frequency of MA use in pregnancy was unknown, but the father described it as "often." The five-year-old girl had diagnoses of congenital nystagmus, pale retinae (ocular albinism), right thalamic infarct in utero with left hemiparesis including the face, cerebral palsy, complex partial epilepsy, attention deficit/hyperactivity disorder with features of opposition defiant disorder and developmental delay. She was documented to have had three episodes of anemia (at nine months, three years and five years of age). The cause of anemia appeared to be uninvestigated. They reported patient medications including carbamazepine for a seizure disorder, methylphenidate for ADHD and a child multivitamin with extra vitamin C. At two years of age, the child was documented as being in the 10th percentile for BMI; by three and a half, she was in the 50th percentile, and by four and a half, she was in the 90th percentile. She reportedly had compulsive eating tendencies. They reported a below normal score of 72 on standardized intelligence age score. Age equivalent scores for gross and fine motor and visual development were reported as being in the range of three years, six months of age. They also observed below-average language skills that included limited vocabulary, but age-appropriate speech production. She obtained high scores in oppositional behavior, cognitive problems and inattention, hyperactivity, social problems, psychosomatic issues and impulsivity.	B<br>C<br>N/I
**Himes SK, LaGasse LL, Derauf C, Newman E, Smith LM, Arria AM, Della Grotta SA, Dansereau LM, Abar B, Neal CR, Lester BM, Huestis MA. Risk of neurobehavioral disinhibition in prenatal methamphetamine-exposed young children with positive hair toxicology results.** ***Ther Drug Monit*****. 2014;*****36*****(4):535–543. DOI: 10.1097/FTD.0000000000000049**<br>The authors investigated the effects of prenatal and postnatal MA exposure on neurobehavioral disinhibition in children 6.5 years old. Mother-infant dyads enrolled in the IDEAL study participated in this investigation. Hair screening was utilized to determine postnatal exposure. Neurobehavioral disinhibition was not significantly different between PME alone and prenatal and postnatal MA exposure combined. They presented similar results for PME and tobacco exposure. But as previous studies have found, they reported that prenatally exposed children showed significant differences in behavioral and executive function in comparison to controls.	B
**Kalaitzopoulos DR, Chatzistergiou K, Amylidi AL, Kokkinidis DG, Goulis DG. Effect of methamphetamine hydrochloride on pregnancy outcome: A systematic review and meta-analysis.** ***J Addict Med.*** **2018;12(3):220–226. DOI: 10.1097/ADM.0000000000000391**<br>They reported a statistically significant decrease in gestational age at birth, neonatal birth weight, head circumference, body length and Apgar score among women that used MA in pregnancy when compared to those who did	M<br>N/I

not. However, they also reported some inconsistencies among studies on gestational age. Based on their results, they found no statistical differences for pre-eclampsia or hypertensive complications when comparing mothers with MA use in pregnancy to mothers without MA use in pregnancy.	
**Kiblawi ZN, Smith LM, Diaz SD, LaGasse LL, Derauf C, Newman E, Shah R, Arria A, Huestis M, Haning W, Strauss A, Della Grotta S, Dansereau LM, Neal C, Lester B. Prenatal methamphetamine exposure and neonatal and infant neurobehavioral outcome: Results from the IDEAL study. *Subst Abus.* 2014;35(1):68–73. DOI: 10.1080/08897077.2013.814614**<br>This study administered the NICU Network of Neurobehavioral Scale (NNNS) to 380 participants gathered for the IDEAL study. This scale was administered at birth and again at one month of age with the intent of studying neurobehavioral outcomes associated with PME. Newborns with PME were characterized as having decreased body lengths and smaller heads. In addition, they presented with a decreased Apgar score at one miniute and were, on average, born prematurely by one week. There were no significant findings for PME alone, however there was a significant interaction between PME and time. At one month old, children with PME showed an increase in arousal and decrease in total stress. Following these changes, children with PME showed no difference from the control group on measurement of arousal and total stress. The authors were unable to explore the association between exposure time and dose with their findings.	N/I
**Kiblawi ZN, Smith LM, LaGasse LL, Derauf C, Newman E, Shah R, Arria A, Huestis M, DellaGrotta S, Dansereau LM, Neal C, Lester B. The effect of prenatal methamphetamine exposure on attention as assessed by continuous performance tests: results from the Infant Development, Environment, and Lifestyle study. *J Dev Behav Pediatr.* 2013;34(1):31–37. DOI: 10.1097/DBP.0b013e318277a1c5**<br>The current study investigated a possible association between PME and attention span. Five-and-half-year-old children from the IDEAL study were assessed for ADHD using the Conners' Kiddie Continuous Performance Test (K-CPT). When comparing neonatal characteristics, infants with PME had a slightly shorter gestational age and shorter birth length than comparison children. Children with PME demonstrated differences in hit reaction time block change and hit standard error interstimulus interval change, measurements defined as vigilance and attention respectively. Children with PME also demonstrated a higher attention-deficit hyperactivity disorder confidence index score than comparisons. However, neither group demonstrated a positive index score, defined as greater than 50%. Analyzing heavy exposure separately did not mitigate the significance of these results. According to their findings they suggest that children of any age with PME can exhibit indicators of risk for ADHD.	B<br>N/I
**Kirlic N, Newman E, Lagasse LL, Derauf C, Shah R, Smith LM, Arria AM, Huestis MA, Haning W, Strauss A, Dellagrotta S, Dansereau LM, Abar B, Neal CR, Lester BM. Cortisol reactivity in two-year-old children prenatally exposed to methamphetamine. *J Stud Alcohol Drugs.* 2013;74(3):447–451. DOI: 10.15288/jsad.2013.74.447**	B<br>C

This study examined two-year-old children with PME from the IDEAL study to investigate a potential association between PME and post-natal environmental stress with cortisol stress reactivity. While they observed both increased and blunted cortisol responses, they identified that children exhibiting an increased cortisol response were associated with higher levels of exposure and greater potential for physical abuse. Those that demonstrated a blunted response were associated with caregiver's postnatal alcohol use, the child's behavioral dysregulation and interaction between higher levels of PME and a post-natal environment characterized by their caregiver's psychopathology. Through these findings, they assert that PME may be associated with changes in the hypothalamic-pituitary-adrenal (HPA) axis reflecting hyperactivity, or in the case of chronic environmental stress such as the examples stated above, hypoactivity.	
**Kwiatkowski MA, Donald KA, Stein DJ, Isper J, Thomas KGF, Roos A. Cognitive outcomes in prenatal methamphetamine exposed children aged six to seven years. *Compr Psychiatry.* 2018;80:24–33. DOI: 10.1016/j.comppsych.2017.08.003**<br>The study was conducted to understand cognitive outcomes of PME in a low-income country. The authors indicated a particular interest in studying cohorts during milestones related to cognitive changes, such as the time children entered formal schooling. In this study, children with PME were ages six to seven, while the control group had slightly older children. While some prenatal drug use was excluded, the inclusion criteria allowed for use of alcohol or nicotine. After accounting for sociodemographic, co-exposure and anthropometric variables, the results showed PME negatively impacted cognitive domains associated with behavioral control, executive function, IQ, cognitive control and sustained attention, learning ability, working memory, verbal and visual memory, confrontation naming ability and visual motor integration. While their results for fine motor coordination were not consistent with other literature, the authors still suggested a potential relationship with PME. The study was limited by a small sample size and poor availability of dosages.	B<br>C
**Kwiatkowski MA, Roos A, Stein DJ, Thomas KG, Donald K. Effects of prenatal methamphetamine exposure: A review of cognitive and neuroimaging studies. *Metab Brain Dis.* 2014;29(2):245–254. DOI: 10.1007/s11011-013-9470-7**<br>This review set out to gather and summarize all literature in neuroimaging, neuropsychology and neuroscience discussing cognitive, behavioral and neuroimaging outcomes associated with PME. They summarized sMRI studies by Change et al (2004) and Sowell et al (2010) that discussed changes in brain volume and a DTI study by Cloak et al (2009) regarding microstructural brain changes. They reviewed one magnetic resonance spectroscopy (MRS) study reported by Smith et al (2001) and Chang et al (2009), and referenced one FMR study which demonstrated effects of PME on a verbal memory task. One study they specifically mentioned was the IDEAL study, which they reported to be the first study to discuss dose-response and trimester-related effects. Few studies have been able to associate structural changes to behavior and condition, but they reported that Chang et al (2004) found a reduction in putamen and globus	B<br>C<br>N

pallidus size in children with PME that was associated with attentional task performance. They also discussed studies reporting metabolic correlation to behavior and cognition.	
**LaGasse LL, Derauf C, Smith LM, Newman E, Shah R, Neal C, Arria A, Huestis MA, DellaGrotta S, Lin H, Dansereau LM, Lester BM. Prenatal methamphetamine exposure and childhood behavior problems at 3 and 5 years of age. *Pediatrics.* 2012;129(4):681–688. DOI: 10.1542/peds.2011-2209**<br>This study examined associations between PME and behavioral problems in children aged three and five. Assessment was done through caregiver reporting using the Child Behavior Checklist (CBCL). In both age groups, children with PME presented more emotionally reactive and anxious/depressed behaviors than their comparisons. Externalizing behavior scores were higher in the five-year-olds with PME but not at three years when compared to their respective control groups. Similarly, children with PME at five years presented higher scores for ADHD issues as compared to the non-exposed group, however this was not observed for children with PME at three years. They also reported that at five years old, children with PME demonstrated less aggressive behavior and higher scores on internalizing scales, anxious/depressed scales, somatic complaints scales and withdrawn scales. Heavy exposure was associated with higher scores for attention problems and being withdrawn than scores from controls. Some/moderate exposure group was associated with higher scores for emotionally reactive than scores from controls. PME was not linked to internalizing or total behavior problem scales.	B<br>C
**LaGasse LL, Wouldes T, Newman E, Smith LM, Shah RZ, Derauf C, Huestis MA, Arria AM, Della Grotta S, Wilcox T, Lester BM. Prenatal methamphetamine exposure and neonatal neurobehavioral outcome in the USA and New Zealand. *Neurotoxicol Teratol.* 2011;33(1):166–175. DOI: 10.1016/j.ntt.2010.06.009**<br>This study examined neurobehavioral changes in children with PME from both the United States (US) and New Zealand (NZ). The participants were enrolled in the IDEAL study conducted in both countries. The NICU Network Neurobehavioral Scale (NNNS) was administered to children within the first five days of life for the purpose of predicting medical and behavioral outcomes. Both cohorts had 'white' as the largest identified racial/ethnic group. The exposed group in US had lower gestational age, were shorter and had smaller head circumferences than controls. These changes were not observed in the NZ cohort. The exposed group in NZ was associated with poorer quality of movement, more total stress/abstinence, more physiological stress and more central nervous system stress than controls. The exposed group in NZ demonstrated more non-optimal reflexes than controls; however, this was not observed in the US cohort. Results from NZ were found to be significantly different from US results on these five scores: increased nonoptimal reflexes, hypotonicity, hypertonicity and less total stress, including CNS stress. Heavy exposure was associated with lower arousal and less excitability than some-or-no-use groups in both countries. MA use during the first trimester in both US and NZ was associated with greater total	N/I

stress/abstinence and physiological stress, while MA use during the third trimester was linked to increased lethargy and hypotonicity.	
**Lester BM, Lagasse LL. Children of addicted women.** ***J Addict Dis.*** **2010;29(2):259-276. DOI: 10.1080/10550881003684921**<br>This review discussed literature on prenatal exposure to cocaine, MA and opiates and the effect exposure had on behavior and development. When discussing PME, they acknowledged findings from the multisite IDEAL study. They reported no differences between groups with PME and controls on the Bayley Scales at ages one, two or three. They referred to a series of studies from Sweden that followed children with PME until age 14 – these studies were published prior to 2010 and as such are not reviewed here. They also reference two neuroimaging studies documented prior to 2010.	C
**Liles BD, Newman E, Lagasse LL, Derauf C, Shah R, Smith LM, Arria AM, Huestis MA, Haning W, Strauss A, Dellagrotta S, Dansereau LM, Neal C, Lester BM. Perceived child behavior problems, parenting stress, and maternal depressive symptoms among prenatal methamphetamine users.** ***Child Psychiatry Hum Dev.*** **2012;43(6):943–957. DOI: 10.1007/s10578-012-0305-2**<br>This study examined maternal depression, parental stress and perceived child behavior problems in mothers who used MA in pregnancy. Although documentation revealed many PME infants who resided with primary caregivers who were not their biological mothers, this study only included biological mothers who had continuous custody of their children from birth to 36 months of age. They found MA use during pregnancy was associated with more depressive symptoms and parenting stress, but no heightened perception of child behavior problems relative to the controls. Regardless of MA exposure, they suggested child behavior problems can predict maternal stress when the child is 36 months old. Depression did not mediate a link between perceived child behavior problems and parenting stress. They reported that depressive symptoms could also stand as a predictor of parenting stress.	B<br>M
**Louw K-A. Substance use in pregnancy: The medical challenge.** ***Obstet Med.*** **2018;11(2):54–66. DOI: 10.1177/1753495X17750299**<br>The author referenced literature that documented a heighted likelihood of growth restriction, ICU admission and difficulty feeding in children with PME. They also recognized data that demonstrated an increased risk of neonatal and infant death in children with PME. They suggested children with PME may be at risk for developmental and behavioral effects, and cognitive problems. They further report that MA use during pregnancy was associated with low birth weight, shorter gestational age, preterm birth, preeclampsia, gestational hypertension, placental abruption and intrauterine death.	B<br>C<br>M<br>N/I
**Madide A, Smith J, Odendaal H. Methamphetamine use by pregnant women: Impact on the neonate and challenges for the perinatal team.** ***Obstet Gynaecol Forum.*** **2012;22(1):8–11. https://www.ajol.info/index.php/ogf/article/view/74784**	B<br>C<br>M<br>N<br>N/I

The authors reported on literature that associated PME with placental abruption, preterm delivery and intrauterine growth restriction with microcephaly, while acknowledging polysubstance use as a potential confounding variable. They referenced a meta-analysis by Ladhani et al. (2011) which reported higher unadjusted odds of preterm birth, SGA and low birth weight with PME. They also referenced literature by Good et al. (2010) who reported that the most common complication in mothers who used MA in pregnancy was preterm delivery. In comparison to non-users, Madide et al also reported higher neonatal mortality and lower Apgar scores. They also reported that a percentage of women who used MA during pregnancy experienced placental abruption and hypertensive complications unrelated to pre-eclampsia. Other literature discussed also observed intrauterine growth restriction in both preterm and term infants. They also referenced findings on dose-related effects, stating that infant exposure throughout pregnancy is associated with lower birth weights and smaller head circumferences than those exposed only in the first two trimesters. They reported on work by Dixon and Bejar (1989) that demonstrated evidence of brain hemorrhage, cavitation and infarction in the basal ganglia, deep in the frontal lobes and the posterior fossa of term infants who were exposed to cocaine and/or MA. The authors of this review suggested that the trend towards small head circumferences may imply poor brain development. They mentioned MRI advances by Derauf et al. (2009), who found significant reductions in subcortical areas like the globus pallidus, putamen and hippocampus. These changes were then associated with deficits of verbal memory and poor sustained attention. Sowell et al. (2010) also utilized MRI methods, but examined differences between MA and alcohol versus alcohol only on developing brain volume. Madide et al commented on the shortage of data regarding frequency and consistency of aberrant neurobehavioral symptoms observed in newborns with PME. But they acknowledge Smith et al.'s (2008) observations of decreased arousal, increased lethargy and physiologic stress associated with heavy MA use during pregnancy. They also reported on several studies that found unexhibited neurobehavioral symptoms in newborns with PME. The authors commented that their facility has observed tremulousness in babies with PME. They drew attention to previous comments on the use of Finnegan's abstinence scoring scheme designed mainly for narcotic exposed newborns, with PME newborns; the comments suggested this scoring may underestimate symptoms experienced by babies with PME. The authors shared that effects of MA use on pregnant woman were similar to effects experienced by others, and included aggressive paranoid psychosis, cognitive impairments, emotional deficits and structural brain changes such as white matter reduction and deep-brain strokes; which may be indirectly similar to altered behavior and below optimal functioning when under the influence of MA. Finally, they suggested that women who use MA during pregnancy may also be indirectly affected by polysubstance use and may potentially be at risk for sexually transmitted diseases, HIV, Hepatitis B and C.	
**Maranella E, Mareri A, Nardi V, Di Natale C, Di Luca L, Conte E, Pannone V, Catalucci A, Di Fabio S. Severe neurologic and hepatic toxicity in a newborn prenatally exposed to methamphetamine. A case report. *Brain Dev.* 2019;41(2):191–194. DOI: 10.1016/j.braindev.2018.08.010**	N/I

This report discussed a severe case of acute neurological and hepatic toxicity in a newborn who experienced PME. Urine screen tests in both mother and newborn returned positive results for MA but no other substance. It is believed that no other substances were used during the pregnancy, including alcohol and tobacco. A few hours after an uncomplicated caesarian delivery, this newborn experienced a severe decline in health. Diagnostic testing identified hepatic insufficiency, coagulopathy, thrombocytopenia and encephalopathy. MRI and cranial ultrasonography identified diffuse white matter damage, as well as two ischemic-hemorrhagic lesions. Follow-up imaging done after one month identified leukomalacia. Three months later, a follow-up identified hypotonia and spontaneous motor activity on the left side of the brain. This case report could be a useful reminder for clinicians to be suspicious of acute neurological and hepatotoxic symptoms that arise in newborns with PME. Further research would be necessary to confirm a relationship between MA and these findings. Other confounding factors would also need to be considered.	
**Maya-Enero S, Candel-Pau J, Rebollo-Polo M, Candela-Canto S, de la Torre R, Lopez-Vilchez MA. Central nervous system malformation associated with methamphetamine abuse during pregnancy. *Clin Toxicol.* 2018;56(8):795–797. DOI: 10.1080/15563650.2018.1428338**<br>This case report discussed the diagnosis of agenesis of foramen of Monro and cortical maldevelopment in a newborn with a Filipina mother who used MA during pregnancy. A physical examination, ultrasound and MRI were instituted to investigate the complex brain malformation. Acute MA use was confirmed through urine screens from both mother and baby. To determine chronic use, maternal hair was screened for MA and results show signs of significant use. MA was found throughout the length of hair, and according to the authors its presence confirms MA use in at least the last two trimesters. Amphetamine was also found in the hair screen.	N/I
**Morie KP, Crowley MJ, Mayes LC, Potenza MN. Prenatal drug exposure from infancy through emerging adulthood: Results from neuroimaging. *Drug Alcohol Depend.* 2019;198:39–53. DOI: 10.1016/j.drugalcdep.2019.01.032**<br>The aim of this review encompasses current literature discussing the influences of prenatal drug exposure. Results were uniquely categorized through a developmental lens separating the stages of life into infancy, childhood and adolescence, and also by imaging modalities. The review went beyond current literature to discuss changes in brain function as opposed to brain development or behavior. The results provided may be of use to researchers interested in expanding their knowledge of this area specifically through imaging methodology. While the review recalls studies discussing several types of substance exposure as well as polysubstance exposure, researchers interested specifically on the influence of MA will also find this review relevant. As an example, they reported that tractography identified reduced white-matter connectivity between multiple brain regions.	N
**Nguyen D, Smith LM, Lagasse LL, Derauf C, Grant P, Shah R, Arria A, Huestis MA, Haning W, Strauss A, Della Grotta S, Liu J, Lester BM. Intrauterine growth of infants exposed to prenatal methamphetamine: results from the infant development, environment, and lifestyle study. *J Pediatr.* 2010;157(2):337–339. DOI: 10.1016/j.jpeds.2010.04.024**	M<br>N/I

Utilizing children enrolled in the IDEAL study, the authors examined the effects of PME on neonatal growth. Mothers who used MA during pregnancy gained more weight than mothers who did not use MA during pregnancy. After comparing weight gain between mothers who used MA in the first or second trimester only to those who used MA in the third as well, those who quit MA earlier gained more weight than those who continued MA use during the third trimester. After adjusting for covariates, MA use during pregnancy was associated with an increased likelihood of the infant being SGA. They also mentioned that in contrast to their previous studies, these results showed no difference in birth weight after adjusting for covariates.	
**Padilla C, Hernandez Conte A, Ramzy D, Lubin L, LaBounty T, Chung JH, Zeng Y. Utilization of "stand-by" extracorporeal membrane oxygenation in a high-risk parturient with methamphetamine-associated cardiomyopathy undergoing dilation and evacuation: A case report. *A A Case Rep.* 2017;8(5):105–108. DOI: 10.1213/XAA.0000000000000441** This case report discussed the utilization of "stand by" extracorporeal membrane oxygenation treatment for an elective dilatation and evacuation of triplet fetuses. The women presented with severe left ventricular dysfunction and previous history for morbid obesity, chronic hypertension and diabetes mellitus type II. The patient was reported to have acute and chronic use of MA. She was also taking antibiotic treatment for a recurring pneumococcal infection. While the patient could have developed MA-associated cardiomyopathy, she had many risk factors that may have also contributed to peripartum cardiomyopathy.	M
**Perumal G, Marathe SP, Schlapbach LJ, Morwood J, Venugopal P, Alphonso N. Postoperative catecholamine resistance following fetal methamphetamine exposure. *Asian Cardiovasc Thorac Ann.* 2019;27(1):30–32. DOI: 10.1177/0218492318784740** This case discussed the cardiovascular involvement and postoperative catecholamine resistance in a newborn boy with PME. The mother disclosed use of MA prenatally, throughout pregnancy, and postnatally while breastfeeding. Following birth, the newborn was diagnosed with a hypoplastic left heart complex and supra mitral membrane. On day 10, he underwent a modified Norwood operation. Within 30 minutes post-operation his catecholamine response declined and a transsternal venoarterial extracorporeal membrane oxygenation (ECMO) was put in place. On day nine of ECMO, catecholamine response returned. This case report identified the potential for cardiovascular complications and catecholamine resistance as outcomes of PME. Different methods of exposure increased uncertainty in the relationship between exposure and diagnostic findings. The mother's demographics as well as potential polysubstance use was not discussed.	N/I
**Pierce SL, Zantow EW, Phillips SD, Williams, M. Methamphetamine-associated cardiomyopathy in pregnancy: A case series. *Mayo Clin Proc.* 2019;94(3):551–554. DOI: 10.1016/j.mayocp.2018.12.008** This series of case reports presented five patients diagnosed with MA-associated cardiomyopathy during pregnancy. Records were collected at the University of Oklahoma Health Sciences Center. The report highlighted some	N/I

variabilities in outcomes but the authors suggested that early medical treatment for MA-associated cardiomyopathy can be reassuring for concerns regarding neonatal complications associated with prematurity. Readers may be cautious of a direct relationship between MA and a diagnosis of cardiomyopathy, with the potential for confounding factors such as family history or a previous diagnosis.	
**Piper BJ, Acevedo SF, Kolchugina GK, Butler RW, Corbett SM, Honeycutt EB, Craytor MJ, Raber J. Abnormalities in parentally rated executive function in methamphetamine/polysubstance exposed children. *Pharmacol Biochem Behav.* 2011;98(3):432–439. DOI: 10.1016/j.pbb.2011.02.013**<br>Children with PME were characterized as being significantly behind similar aged children in school. Children with PME were twice as likely to be behind classmates in reading and were four-fold more likely of having an diagnosis of ADHD. Children with PME exhibited pronounced dysfunction in executive function and a small deficit in spatial memory. Global Executive Composite and Behavioral Regulation and Metacognition Indexes were significantly different among children with PME. Although changes in the Global Executive Composite scores were independent of the rater (biological or non-biological primary caregiver), the authors identified some evidence indicating the executive function profile was more concerning in those children whose raters were non-biologically related. They suggested that children placed in adoptive care might experience early developmental history more severely. Another possibility they suggested is that non-biological caregivers may be more extreme in their ratings than biological parents. Several behavioral assessments were suspected to be altered such as IQ, Spatial Span, Family Pictures, Dot Location and vigilance performance, but were found to be unaffected by PME. Based on these results, the authors suggested PME was associated with a selective profile of abnormalities in parentally rated executive function.	B<br>C
**Roos A, Donald KA. Refining the understanding of the effects of prenatal methamphetamine and tobacco exposure on the developing brain. *JAMA Psychiatry,* 2016;73(12):1228–1229. DOI: 10.1001/jamapsychiatry.2016.2845**<br>This article discussed the influence of PME and tobacco on the developing brain of the fetus. The article cited very few references. In the discussion about MA, it relies almost entirely on one article by Chang et al. (2016) that discussed white matter microstructure and neurological development in infants prenatally exposed to MA and/or tobacco.	N
**Roos A, Jones G, Howells FM, Stein DJ, Donald KA. Structural brain changes in prenatal methamphetamine-exposed children. *Metab Brain Dis.* 2014;29(2):341–349. DOI: 10.1007/s11011-014-9500-0**<br>This study set out to investigate changes that might occur to brain volume and cortical thickness in children with PME. Unlike the IDEAL study where prospective data was able to be collected, this study was limited to the knowledge available six years after the prenatal exposure. The two variables were assessed using Freesurfer. In comparison to controls, they found that left putamen volume was significantly increased in children with PME. Children with PME presented with a reduced left hemisphere cortical	N

thickness of the inferior parietal, parsopercularis and precuneus areas as compared to the controls. The authors also commented on gender dependent effects: in comparison to male controls, males with PME had greater volumes in striatal and associated regions (left globus pallidus and left and right ventral diencephalon). Males with PME also had greater a volume in the right ventral diencephalon volume as compared to females with PME; however the opposite relationship was found between control males and control females. In addition, females with PME presented with greater cortical thickness in the banks of the superior central sulcus and cuneus as well as a reduced mid-posterior corpus callosum volume, as compared to control females.	
**Roos A, Kwiatkowski MA, Fouche JP, Narr KL, Thomas KG, Stein DJ. White matter integrity and cognitive performance in children with prenatal methamphetamine exposure. *Behav Brain Res.* 2015;279:62–67. DOI: 10.1016/j.bbr.2014.11.005**<br>This study utilized DTI to examine changes in the brain's white matter integrity and its associated cognitive performance in children with PME. Previous studies explored the possibility of these changes; however, the current literature showed inconsistent changes among DTI parameters. To improve study findings, the researchers utilized a narrow age group to minimize differences potentially arising due to timing in development and the effects of PME. They also accounted for nicotine use and gender differences during analysis. Results indicated changes in the white matter of children with PME as compared to the control group. These changes were associated with changes in motor coordination, cognitive ability and executive function. No differences were found in anthropometric measures between the children with PME and controls. These results are useful to researchers and clinicians interested in the cognitive changes seen among children with PME. There might be concerns with the validity of the cognitive tests performed on non-English speaking children, but the researchers addressed this through the use of the non-verbal scale of Kaufman Assessment Battery for Children – II with this population since language and culture was different.	N/I
**Ross EJ, Graham DL, Money KM, Stanwood GD. Developmental consequences of fetal exposure to drugs: What we know and what we still must learn. *Neuropsychopharmacology.* 2014;40:61–87. DOI: 10.1038/npp.2014.147**<br>The authors referred to several studies which identified that PME is commonly associated with growth restriction, decreased weight, length and head circumference in newborns. They gave attention to Zabeneh et al.'s (2012) work which identified a modest decrease in height but not weight, head circumference, or weight-for-length trajectories in in the first three years of life in children with PME. In accordance with Derauf et al. (2012) and Zabeneh et al. (2012), PME was associated with reduced volume in the caudate nucleus and increased cortical thickness in perisylvian and orbital frontal cortices. DTI work by Cloak et al. (2009) reported lower diffusion and higher fractional anisotropy in children with PME at ages three to four. They also reported on Chang et al. (2009) who studied metabolic changes via magnetic resonance spectroscopy (MRS). Another MRI study reported lower scores on visual motor, integration, attention, verbal memory and long-term spatial memory. Smith et al. (2011) reported poorer performance in fine motor performance	B<br>C<br>N<br>N/I

during the first year of life in children with PME as compared to controls, but this seemed to resolve by three years of age. The IDEAL study revealed PME was associated with child externalizing behavioral problems at five years of age. They also associated parenting stress and psychological symptoms of the primary caregiver to behavioral problems in the child. Lastly, the authors reference a cross-national study on the effects of prenatal use in New Zealand and the United States. Effects appeared to be different across countries (Abar et al, 2013).	
**Roussotte FF, Bramen JE, Nunez SC, Quandt LC, Smith L, O'Connor MJ, Bookheimer SY, Sowell ER. Abnormal brain activation during working memory in children with prenatal exposure to drugs of abuse: the effects of methamphetamine, alcohol, and polydrug exposure. *NeuroImage.* 2011;54(4):3067–3075. DOI: 10.1016/j.neuroimage.2010.10.072**<br>Children ranging from seven to 15 years of age were categorized into groups based on exposure, including PME with alcohol exposure, alcohol exposed only and a non-exposed control group. fMRI was utilized for studying functional alterations in the fronto-striatal circuit that were observed during a visuospatial working memory (WM) "N-Back" task. MA exposure was associated with less activation in brain areas including the frontal and basal ganglia regions in the left hemisphere during working memory, with the most prominent differences observed in the left caudate, left putamen and left inferior frontal gyrus, around Broca's area, as compared to unexposed controls. Other brain regions located in the left hemisphere, including the middle frontal gyrus, the precentral gyrus, the frontal orbital cortex, the superior and middle temporal gyri, the temporal pole, the palnum temporale and insula, were associated with decreased activation in the children with PME in comparison to controls. In comparison to controls, children with PME revealed reduced recruitment of left and right thalamus during working memory versus rest. The authors negatively correlated performance and activation in the inferior temporal gyrus and temporal pole, the iddle temporal gyrus, the anterior cingulate and paracingulate gyri, the frontal orbital cortex and frontal pole,and the left superior frontal gyrus in children with MA exposure. PME also revealed a negative correlation between performance and activation in the left parahippocampal gyrus, and bilaterally in the pre- and post-central gyri, superior temporal gyrus and putamen. As a result of these findings, the authors suggested PME was linked to structural damage in the fronto-striatal circuit which then lends to decreased recruitment of this circuit during working memory.	N
**Roussotte FF, Rudie JD, Smith L, O'Connor MJ, Bookheimer SY, Narr KL, Sowell ER. Frontostriatal connectivity in children during working memory and the effects of prenatal methamphetamine, alcohol, and polydrug exposure. *Dev Neurosci.* 2012;34(1):43–57. DOI: 10.1159/000336242**<br>They reported that, compared to a cohort without PME, children/adolescents with PME showed increased connectivity between the putamen seeds and frontal brain, while caudate seeds showed fewer negative correlations, particularly in the occipital regions. As hypothesized, connection	C<br>N

between the dorsal caudate and frontal executive network was reduced in the MA exposed group.	
**Roussotte F, Soderberg L, Sowell E. Structural, metabolic, and functional brain abnormalities as a result of prenatal exposure to drugs of abuse: evidence from neuroimaging. *Neuropsychol Rev.* 2010;20(4):376–397. DOI: 10.1007/s11065-010-9150-x**<br>The article reviewed current neuroimaging literature focused on the metabolic, structural, and functional changes that occur in a developing brain prenatally exposed to alcohol, cocaine and MA. The authors acknowledged a few large prospective studies which have documented restricted fetal growth and poorer neurobehavioral outcomes such as reduced arousal and depressed movement scores. They reference one study by Sowell et al. (2010) that utilized sMRI to differentiate effects of PME from alcohol exposure. Sowell et al.'s (2010) findings suggested that striatal and limbic structures were especially vulnerable to the effects of MA. They reported only one DTI study which found lower Apparent Diffusion Coefficient (ADC) in the frontal and parietal white matter when PME is compared to controls. A recent magnetic resonance spectroscopy (MRS) study detected metabolic abnormalities in the frontal white matter that is potentially indicative of abnormal neuronal and glial development. A few of the imaging studies mentioned made further correlations to neurocognitive performance in children with PME. Chang et al. (2009) associated reduced MI concentrations in the thalamus with poor performance on a visual motor integration task; while Sowell et al. (2010) reported volume reductions in the caudate correlated with full-scale intelligence quotient (FSIQ) scores. The authors reported only one fMRI study that found more diffuse activation of brain regions important for verbal memory.	B<br>C<br>N<br>N/I
**Sakai K, Iwadate K, Maebashi K, Matsumoto S, Takasu S. Infant death associated with maternal methamphetamine use during pregnancy and delivery: A case report. *Leg Med.* 2015;17(5):409–414. DOI: 10.1016/j.legalmed.2015.06.004**<br>This case report presented the delivery of a presumed stillborn infant to a mother who used MA in pregnancy. They asserted that this case presented the highest blood concentration of MA recorded in fatal infant intoxication. This MA concentration led them to believe there was a strong likelihood the death was caused by MA intoxication. However, it was not known whether concentrations could be altered by redistribution. Furthermore, there was no evidence of histopathological findings that previously have been reported in association with stimulant intoxication.	N/I
**Shah R, Diaz SD, Arria A, LaGasse LL, Derauf C, Newman E, Smith LM, Huestis MA, Haning W, Strauss A, Della Grotta S, Dansereau LM, Roberts MB, Neal C, Lester BM. Prenatal methamphetamine exposure and short-term maternal and infant medical outcomes. *Am J Perinatol.* 2012;29(5):391–400. DOI: 10.1055/s-0032-1304818**<br>This study explored short term outcomes in mother and baby following MA use in pregnancy. They found mothers who used MA in pregnancy were more likely to be diagnosed with gonorrhea or a psychiatric disorder/emotional illness than comparison women. Several other conditions previously associated with MA use in pregnancy were not significant. They suggested previous studies may not have properly controlled for covariates. The authors emphasized the prevalence of maternal complications/conditions that had	M<br>N/I

more than five cases. Infants with PME were associated with poor sucking reflex, admission to the NICU and the involvement of child and protective services. Infants with PME were also less likely to be breast-fed. They also reported that newborns with PME have smaller head circumferences and length.	
**Smid, M. Methamphetamines, mamas and munchkins--the drug epidemic we aren't talking about. [Addiction Technology Transfer Center Network website]. 2017. https://attcnetwork.org/media/1226 Accessed May 29, 2020.**<br>Smid discussed MA and pregnancy being associated with severe preeclampsia, intrauterine growth restriction (IUGR), maternal cardiac problems/pulmonary edema, abruption (but more commonly associated with cocaine) and preterm labor. She presented statistical results associating MA use in pregnancy with gestational age, chronic hypertension and cesarian delivery. She suggested sex differences linked with MA exposure may be observable in the fetal period. These changes could include male offspring having an increased risk for drug induced neurotoxicity as adults, or female children having changes in the frontal white matter that could be suggestive of neuronal and glial development. Using results from the IDEAL study, she identified that MA use in pregnancy has been associated with increased likelihood for admittance to the NICU, decreased arousal and increased physiological stress, which improved by one month of age. At ages three and five, heavy PME was linked to increased anxiety/depression and attention problems. At age seven and half, children with PME had lower cognitive function as measured by the Conner's Parent Rating Scale. Smid discussed three traditional approaches to addiction in pregnant women. The first included involving CPS, suggesting the mother is unfit to parent with an addiction. The second approach involved arresting the woman, reasoning that she would stay clean in jail. The third approach was similar; again, arresting the woman but instead justifying this choice with the reasoning that she could be forced into enrollment in a drug treatment program as a condition of discharge. Alternatively, Smid identified non-traditional interventions that are family-oriented and gender-specific. She acknowledged novel approaches by the Clinical & Translational Science Award Program's pregnancy and opioids models of care (PROMO) study models, University of North Carolina's Horizons Clinic, and University of Hawaii's path as examples of alternative approaches.	B<br>C<br>I<br>M<br>N/I
**Smid MC, Metz TD, Gordon AJ. Stimulant use in pregnancy: An under-recognized epidemic among pregnant women. *Clin Obstet Gynecol.* 2019;62(1):168–184. DOI: 10.1097/GRF.0000000000000418**<br>The purpose of this review was to provide a summary of perinatal, maternal and child outcomes associated with stimulant use in pregnancy. The authors identified the pharmacology, pathophysiology and epidemiology for several stimulants. Those interested in the use of these stimulants and their effects during pregnancy will find this review helpful. Those solely interested in MA outcomes will find a specific subsection dedicated to the discussion of perinatal effects, including neonatal death, congenital anomalies and neonatal symptoms, as well as maternal and child outcomes associated with PME.	N/I

**Smith LM, Diaz S, LaGasse LL, Wouldes T, Derauf C, Newman E, Arria A, Huestis MA, Haning W, Strauss A, Della Grotta S, Dansereau LM. Developmental and behavioral consequences of prenatal methamphetamine exposure: A review of the Infant Development, Environment, and Lifestyle (IDEAL) study. *Neurotoxicol Teratol.* 2015;51:35–44. DOI: 10.1016/j.ntt.2015.07.006**<br>This was a review of the IDEAL study. The IDEAL study was a seven and a half year longitudinal, multi-centred, case-control study of children with PME and their mothers. The review stressed the importance of presenting findings in the contexts of both the home environment and characteristics of primary caregiver. Results appeared to confirm the influence of these contexts on child outcomes. While mothers who used MA also used other substances, the authors indicated that multivariate statistical analysis controlled for these other variables. No differences were found in maternal complications or newborn health outcomes. Neonatal abstinence syndrome was not observed in infants with PME. Although somatic growth was significantly decreased in children with PME, growth inconstancies were reported across sites. The authors suggested this may be influenced by a difference in policies for maternal drug use between the United States and New Zealand. Heavy drug use was associated with increased stress responses, poorer fine motor scores, and inhibitory controlin the neonate.	C<br>M<br>N/I
**Smith LM, LaGasse LL, Derauf C, Newman E, Shah R, Haning W, Arria A, Huestis M, Strauss A, Della Grotta S, Dansereau LM, Lin H, Lester BM. Motor and cognitive outcomes through three years of age in children exposed to prenatal methamphetamine. *Neurotoxicol Teratol.* 2011;33(1):176–184. DOI: 10.1016/j.ntt.2010.10.004**<br>In this study, motor and cognitive development were assessed in children with PME at ages one, two and three. The cohort included children enrolled in the IDEAL study. The authors reported no differences in gender, birth weight, incidence of SGA, or low birth weight among children with PME, but did report decreased gestational age, length and head circumference at birth among this group. Cognition was assessed using the Bayley Scales of Infant Development (BSID-II), which included mental and motor scales presented as Mental Developmental Index (MDI) and Psychomotor Development Index (PDI) respectively. Peabody Developmental Motor Scales (PDMS-2) administered at one- and/or three-year visits were used to assess gross and fine motor skills. At one year, grasping scores in children with PME were significantly lower than controls, reflecting a certain deficit in fine motor coordination (PDMS-2); however this was not seen at three years of age. Children with heavy PME demonstrated lower grasp scores than children with some-or-no MA exposure, which the authors suggested was attributable to a dose-response effect. Again, at three years, no effects were observed; however children with heavy PME demonstrated lower grasping scores. Longitudinal analysis revealed lower grasping scores with any MA exposure that persisted until three years of age. No effects of MA exposure were observed on MDI and PDI.	C<br>N/I
**Smith LM, Paz MS, LaGasse LL, Derauf C, Newman E, Shah R, Arria A, Huestis MA, Haning W, Strauss A, Della Grotta S, Dansereau LM, Neal C,**	N/I

**Lester BM. Maternal depression and prenatal exposure to methamphetamine: neurodevelopmental findings from the infant development, environment, and lifestyle (ideal) study. *Depress Anxiety.* 2012;29(6):515–522. DOI: 10.1002/da.21956**<br>These researchers were interested in characterizing the effects of maternal depression and methamphetamine exposure on neurobehavioral outcomes. Findings reported from the IDEAL study revealed that infants with PME were more likely to have a lower five-minute Apgar score than comparisons. The researchers associated PME with increased arousal and handling and decreased ability to self-regulate. Maternal depression was found to be associated with increased autonomic stress and decreased quality of movement. They concluded that maternal depression can influence neurobehavioral outcomes in infants as young as one month old, regardless of PME status.	
**Smith LM, Santos LS. Prenatal exposure: The effects of prenatal cocaine and methamphetamine exposure on the developing child. *Birth Defects Res C: Embryo Today.* 2016;108(2):142–146. DOI: 10.1002/bdrc.21131**<br>The authors discussed a report by Shah et al. (2012), who found no difference in incidence of facial dysmorphism, skeletal or cardiac defects or respiratory problems between infants with PME and infants with no exposure. Since there were no signs or symptoms suggestive of drug withdrawal syndrome requiring pharmacological intervention, they suggested that there was no neonatal abstinence syndrome associated with MA exposure. They reported on Zabaneh et al's (2012) findings that, by three years of age, children with PME remained significantly shorter than those without PME. The authors proposed that since fetal growth restriction has been linked to metabolic syndrome and obesity, children with PME could be at a heighted risk for these diseases. They discussed alterations to behavior and referred to LaGasse et al. (2011) who reported that newborns with PME exhibited disorganized state behaviors with poorer quality of movement, increased physiological stress, total stress/abstinence and CNS stress. They also discussed Kiblawi et al (2014), who reported that disorganized behaviors resolved by one month of age. Relying on evidence by Smith et al. (2015), they reported that children with PME showed no deficits in IQ or language development. Instead, the authors reported that PME is associated with increased anxious/depressed problems and emotional reactivity (LaGasse et al., 2012), alongside externalizing behaviors and ADHD symptoms. They also reported on an ADHD confidence index score that was suggestive of a heightened risk for developing ADHD (KIblawi et al., 2013). Based on Exe et al. (2015), they reported that children aged seven and a half with PME demonstrated higher scores for externalizing behaviors such as aggression and rule breaking based on the Child behavior checklist. They also reported the combination of heavy PME and heightened childhood adversity was associated with poorer inhibitory control (Derauf et al., 2012) and overall neurodevelopmental function (Abar et al., 2013). Lastly, they found that greater parental monitoring and higher home life scores decreased the potential for adverse behaviors.	B<br>C<br>I<br>N/I
**Smith ME. Caring for children with prenatal substance exposure: An educational video and pilot study [Doctoral dissertation]. The University of**	B<br>C

**Arizona; 2017. ProQuest Dissertations & Theses Global. https://repository.arizona.edu/handle/10150/626704.**<br>To create an informative video, the author reviewed current literature regarding the effects of PME. They reported that several neuroimaging studies identified alterations in the brain, including reduced volume in the caudate nucleus and hippocampus. They suggested these changes in the hippocampus might provide insight to deficits in attention, memory tasks and behavior reported in children with PME. One MRS study identified reduced levels of myoinositol in the thalamus, which they suggested could be correlated with poor performance on visual motor integration tasks in preschoolers with PME. The majority of evidence specific to the outcomes in infancy, school-age and adolescence were obtained from the IDEAL study.	N
**Sowell ER, Leow AD, Bookheimer SY, Smith LM, O'Connor MJ, Kan E, Rosso C, Houston S, Dinov ID, Thompson PM. Differentiating prenatal exposure to methamphetamine and alcohol versus alcohol and not methamphetamine using tensor-based brain morphometry and discriminant analysis. *J Neurosci.* 2010;30(11):3876–3885. DOI: 10.1523/JNEUROSCI.4967-09.2010**<br>This study analyzed MRI data using tensor-based morphometry (TBM) to investigate alterations in brain volume associated with PME. The study groups were: 1) PME accompanied by alcohol exposure, 2) alcohol without MA exposure, and 3) controls. Both groups with exposures demonstrated reduced volume in thalamic and striatal structures bilaterally, left parieto-occipital and right anterior prefrontal cortices as compared to controls. In contrast to the controls, both groups with exposures demonstrated an increase in volume in bilateral limbic cortices of the anterior and posterior cingulate, as well as in the ventral and medial temporal lobes and bilateral perisylvian cortices. Utilizing statistical comparison, they found that group 2 (PME only) demonstrated more severe volume reductions in the striatum than group 1 (PME and alcohol), in comparison to group 3. Group 2 also demonstrated a great volume increase in the anterior and posterior cingulate regions and the left and right perisylvian cortices as compared to group 1, and in comparison to controls. PME was also associated with a negative correlation between full-scale intelligence quotient (FSIQ) scores and caudate volume.	N
**The Royal Women's Hospital. Drug and Alcohol – Management of Methamphetamine Dependence in Pregnancy [Guideline]. 2017 May 16. https://thewomens.r.worldssl.net/images/uploads/downloadable-records/clinical-guidelines/drug-and-alcohol-management-methamphetamine-dependence-in-pregnancy_160517.pdf Accessed July 21, 2020.**<br>The authors report that PME is associated with a risk of preterm birth and intrauterine growth restriction (IUGR). They also commented that neonatal abstinence syndrome was observed in infants with PME. At admission, they informed women that current literature has associated MA use in pregnancy with associated risks of preterm labour and birth, low birth weight and SGA. On discharge, they counseled mothers on the dangers of using drugs, alcohol, tobacco and sharing sleeping surfaces with their baby to reduce risks of sudden infant death syndrome.	I<br>N/I

**Tsai S-YA, Bendriem RM, Lee C-TD. The cellular basis of fetal endoplasmic reticulum stress and oxidative stress in drug-induced neurodevelopmental deficits.** ***Neurobiol Stress.*** **2019;10. DOI: 10.1016/j.ynstr.2018.100145**<br>The authors of this review referenced Sowell et al.'s (2010) findings on striatal volume reductions in children with PME, which was linked to cognitive deficits. Roos et al. (2014) added to this literature reporting enlargement of the putamen with PME. The reviewers suggested a potential pattern of abnormal growth, followed by slowed growth and impaired brain development in children with PME.	N
**Twomey J, LaGasse L, Derauf C, Newman E, Shah R, Smith L, Arria A, Huestis M, DellaGrotta S, Roberts M, Dansereau L, Neal C, Lester B. Prenatal methamphetamine exposure, home environment, and primary caregiver risk factors predict child behavioral problems at 5 years.** ***Am J Orthopsychiatry.*** **2013;83(1):64–72. DOI: 10.1111/ajop.12007**<br>These researchers wanted to determine if there was an association between PME, home environment, primary caregiver psychological characteristics and the risk factors on developing child behaviors. Among five-year old children from the IDEAL study, PME was associated with internalizing or total behavioral problems. Home environments that provided developmental stimulation and emotional responsiveness decreased risk for internalizing and externalizing behavioral problems. Primary caregivers' parenting stress was linked to a threefold increase in child internalizing behavioral problems. The authors noted that raising a child with PME and behavioral problems can adversely affect parenting stress. Caregivers' psychological symptoms were predictive of an increased likelihood for child externalizing problems and total behavioral problems. The authors reported that older age of primary caregivers was associated with a decreased risk for internalizing, externalizing and total behavioral problems. They suggested that total behavioral problems experienced by children with PME were associated with increased difficulties navigating academic and social demands. Children with PME experiencing increased externalizing behavior might present with deficits in inhibitory control, with the possibility of executive function domains being affected as well. These deficits in executive function may be more precisely noted via challenges in self-regulation, impulsivity and social relationships.	B
**Warton FL, Meintjes EM, Warton CMR, Molteno CD, Lindinger NM, Carter RC, Zollei L, Wintermark P, Jacobson JL, van der Kouwe A, Jacobson SW. Prenatal methamphetamine exposure is associated with reduced subcortical volumes in neonates.** ***Neurotoxicol Teratol.*** **2018;65:51–59. DOI: 10.1016/j.ntt.2017.10.005**<br>This study discussed the impacts of PME on newborn brain volume. Mothers were recruited in pregnancy and newborns were screened in the first month of life. The study included 18 newborns with PME, and twenty-one newborns without PME as controls. The study included food security and caloric intake in their demographic measures. MRI, manual segmentation of subcortical and cerebellar regions and Pearson Correlation analysis were utilized to investigate a possible relationship between volumetric brain	N<br>N/I

changes and PME. Results showed that an increase in PME was correlated to reduction in the right caudate volume and a decrease in the left caudate and bilateral thalamus at trend level. After accounting for confounding factors, the right caudate changes remained significant, however the left caudate and bilateral thalamus remained at trend level. The study also found lower gestational ages at delivery and an increased risk of intrauterine passing of meconium among the cohort with PME. This study included controls that smoked cigarettes, used marijuana and drank alcohol.	
**Warton FL, Taylor PA, Warton CMR., Molteno CD, Wintermark P, Lindinger NM, Zollei L, van der Kouwe A, Jacobson JL, Jacobson SW, Meintjes EM. Prenatal methamphetamine exposure is associated with corticostriatal white matter changes in neonates. *Metab Brain Dis.* 2018;33(2):507–522. DOI: 10.1007/s11011-017-0135-9**<br>The purpose of this study was to investigate possible changes in white matter microstructure within the striatal and orbitofrontal regions of infants with PME. Mothers were recruited antenatally and DTI studies were performed within the first month of life. Results showed reduced fractional anisotropy (FA) and increased radial diffusivity (RD) in white matter connections that may be associated with cognitive dysfunction. A limitation to this study was that mothers who used other substances such as marijuana and alcohol or smoked were included in the control group.	N
**Wouldes T. Prenatal exposure to methamphetamine: A tale of two cultures! [AOD Provider Collaborative website]. N.D. http://www.aodcollaborative.org.nz/vdb/document/36**<br>The article reported findings from the IDEAL study that in the US, 40% of referrals to child protective services (CPS) were specifically related to drug concerns, while in New Zealand (NZ), 15% of referrals were for drug concerns alone. White Europeans were reportedly the largest ethnicity group who self-identified with MA use during pregnancy. The article acknowledged findings from Lagasse, Wouldes et al. (2011) which reported that at one month of age, infants in NZ with PME had more asymmetric reflexes than infants without PME. The report provided information on mothers in NZ who only used MA and were 5.5 times more likely to encounter comorbid diagnosis of substance use disorder (SUD) and psychiatric disorder. Therefore, it is suggested that mothers be treated for both substance use and mental illness. More robust results in infants with PME in NZ around early motor development encouraged more early interventions to treat children with PME.	M<br>N/I
**Wouldes TA, LaGasse LL, Derauf C, Newman E, Shah R, Smith LM, Arria AM, Huestis MA, DellaGrotta S, Wilcox T, Neal CR Jr, Lester BM. Co-morbidity of substance use disorder and psychopathology in women who use methamphetamine during pregnancy in the US and New Zealand. *Drug Alcohol Depend.* 2013;127(1-3):101–107. DOI: 10.1016/j.drugalcdep.2012.06.016**<br>This study examined women who used MA during pregnancy and one month post-partum to determine the co-morbidity of substance use disorders (SUD) and psychiatric disorders. The probability of a SUD was determined using the Substance Abuse Subtle Screening Inventory-3, while psychiatric	M

disorders were identified via the Brief Symptom Inventory (BSI). Women were recruited through the cross-national IDEAL study. Mothers who used MA during pregnancy in both the United States (US) and New Zealand (NZ) showed a 10-fold increase for a SUD and were twice as likely to be diagnosed with a psychiatric disorder,compared to mothers without SUD. The authors reported risk for co-morbid SUD and a positive diagnosis for a psychiatric disorder to be heightened five-fold among mothers in NZ who used MA, but not among mothers in US. They suggested this difference could be attributed to the amount of alcohol consumed by mothers in NZ, which might lead to psychiatric distress and a higher probability for a SUD.	
**Wouldes TA, Lagasse LL, Huestis MA, Dellagrotta S, Dansereau LM, Lester BM. Prenatal methamphetamine exposure and neurodevelopmental outcomes in children from 1 to 3 years. *Neurotoxicol Teratol.* 2014;42:77–84. DOI: 10.1016/j.ntt.2014.02.004**<br>This study explored the neurodevelopment effects of PME in children aged one to three among New Zealand participants from the IDEAL study. The authors utilized the Mental Developmental Index (MDI) and the Psychomotor Developmental Index (PDI) of the Bayley Scales of Infant Development, Second Edition (BSID-II) to measure cognitive and motor performances at ages one, two and three. The Peabody Developmental Motor Scale, Second Edition (PDMS-2) was used to measure gross and fine motor performances at ages one and three. By measuring the child's environment with the Home Observation of Measurement of the Environment and the Maternal Lifestyle Interview, they were able to consider how the environment might influence their observations. In addition to the environmental variables, they utilized the Substance Use Inventory (SUI) to quantify dose and frequency of prenatal drug use across four time periods. After comparing characteristics between the two groups, they found that children with PME had smaller heads at one year, weighed less at two years and were shorter at three years. They also reported that children with PME had poorer psychomotor scores on the BSID-II at one and two years of age. Longitudinal models exploring the effects of PME over the first three years of age demonstrated lower scores on composite measures of psychomotor performance (PDI) and gross motor performance on the PDMS-2. They found no association between exposure and cognitive scores on the BSID-II or fine motor development on the PDMS-2. Like other studies in the field, their results suggested a mediating role between gender and PME outcomes such that males generally demonstrated poorer scores on fine motor tasks as compared to their female counterparts.	C<br>N/I
**Wouldes TA, Lester BM. Stimulants: How big is the problem and what are the effects of prenatal exposure? *Semin Fetal Neonatal Med.* 2019;24(2):155–160. DOI: 10.1016/j.siny.2019.01.011**<br>The intent of this review was to discuss the global rise of stimulant use, particularly cocaine and MA, among child-bearing women. The authors discussed social, biological and psychological factors that might be contributing to the rising prevalence of stimulant use among this population. They suggested there were no consistent findings of neonatal abstinence syndrome requiring pharmacological treatment for cocaine or MA exposure. Instead, they suggested that individually and together, cocaine and MA	B<br>N/I

exposure in utero was associated with disorganized neurobehavior. The second focus of this review was to identify the outcomes of stimulant use in pregnancy and how to better identify and treat women using stimulants while pregnant.	
**Wright TE, Schuetter R, Tellei J, Sauvage L. Methamphetamines and pregnancy outcomes.** ***J Addict Med.*** **2015;9(2):111–117. DOI: 10.1097/ADM.0000000000000101**<br>This study reported on the largest cohort study on PME, which collected prospective data on prenatal MA and other drug use, and pregnancy outcomes. They identified 144 infants with PME and categorized them according to the use in each trimester. The PME group was compared to non-exposed and non-drug exposed groups of infants. The results showed infants with PME had a smaller mean birth weight than the non-drug exposed infants, while non-exposed infants were similar to infants with PME. Infants with positive meconium or urine toxicology at birth were smaller than those exposed only in the first trimester. Gestational age was significantly shorter in the PME group than among both non-exposed and non-drug exposed infants. The non-drug exposed infants had greater head circumferences and were longer. Quitting MA use anytime during the pregnancy improved outcomes such as gestational age and weight. When controlling for shorter gestational age, infants with PME were not smaller. This result stands in contrast to the IDEAL study in which MA-exposed infants were reported to have an increased likelihood of being small for gestational age (SGA).	N/I
**Zabaneh R, Smith LM, LaGasse LL, Derauf C, Newman E, Shah R, Arria A, Huestis M, Haning W, Strauss A, Della Grotta S, Dansereau LM, Lin H, Neal C, Lester BM. The effects of prenatal methamphetamine exposure on childhood growth patterns from birth to 3 years of age.** ***Am J Perinatol.*** **2012;29(3):203–210. DOI: 10.1055/s-0031-1285094**<br>The article examined growth parameters in children with PME from birth to age three. The cohort included children enrolled in the IDEAL study. Adjustments were made for preterm infants as well as other covariates. The authors found no differences in weight, head circumference and weight-for-length growth trajectories between children with PME and the comparison group. Term infants with PME had a shorter height trajectory over the first three years following birth, but by age three, the growth rate per month was similar between PME and control groups. The authors reported no differences in incidence of SGA or birth weight less than 2500 grams, newborn gender and birth weight between PME and control groups. However, they did mention that the full follow-up sample showed an increased prevalence of SGA among those with PME as compared to the controls. They also noted maternal weight gain was significantly higher among those with PME.	M<br>N/I
**Zuloaga DG, Jacosbskind JS, Raber J. Methamphetamine and the hypothalamic-pituitary-adrenal axis.** ***Front Neurosci.*** **2015;9:178. DOI: 10.3389/fnins.2015.00178**<br>The article acknowledged Kirlic et al.'s (2013) findings that children with PME experienced higher stress-induced cortisol levels. They discussed that behavioral effects that have previously been documented in association	C<br>N<br>N/I

with PME such as altered infant arousal, increased emotional reactivity and anxiety/depressed behavior in three- to five-year-olds may be associated with alteration in the hypothalamic-pituitary-adrenal axis.	

[1] B = Behavioral; C = Cognitive; I = Intervention; M = Maternal; N = Neurological/neuroimaging; N/I = Neonatal/Infant

List of common abbreviations used in Annotations:

ADHD: Attention-deficit/hyperactivity disorder
CPS: Child protective services
DTI: Diffusion tensor imaging
HIV: Human immunodeficiency virus
IDEAL: Infant Development, Environment, and Lifestyle
IQ: Intelligence quotient
MA: Methamphetamine
MRI/FMRI/sMRI: Magnetic resonance imaging/functional magnetic resonance imaging/ structural magnetic resonance imaging
NICU: Neonatal intensive care unit
NZ: New Zealand
PME: Prenatal methamphetamine exposure
SGA: Small for gestational age
US/USA: United States of America
